# Cost-competitive decentralized ammonia fertilizer production can increase food security

**DOI:** 10.1038/s43016-024-00979-y

**Published:** 2024-05-16

**Authors:** Davide Tonelli, Lorenzo Rosa, Paolo Gabrielli, Alessandro Parente, Francesco Contino

**Affiliations:** 1 Institute of Mechanics, Materials and Civil Engineering, UCLouvain, Ottignies-Louvain-la-Neuve, Belgium; 2 Aero-Thermo-Mechanics Department, ULB, Brussels, Belgium; 3 Department of Global Ecology, Carnegie Institution for Science, Stanford, CA USA; 4 Institute of Energy and Process Engineering, ETH Zurich, Zurich, Switzerland

**Keywords:** Climate-change mitigation, Agriculture, Environmental studies

## Abstract

The current centralized configuration of the ammonia industry makes the production of nitrogen fertilizers susceptible to the volatility of fossil fuel prices and involves complex supply chains with long-distance transport costs. An alternative consists of on-site decentralized ammonia production using small modular technologies, such as electric Haber–Bosch or electrocatalytic reduction. Here we evaluate the cost-competitiveness of producing low-carbon ammonia at the farm scale, from a solar agrivoltaic system, or using electricity from the grid, within a novel global fertilizer industry. Projected costs for decentralized ammonia production are compared with historical market prices from centralized production. We find that the cost-competitiveness of decentralized production relies on transport costs and supply chain disruptions. Taking both factors into account, decentralized production could achieve cost-competitiveness for up to 96% of the global ammonia demand by 2030. These results show the potential of decentralized ammonia technologies in revolutionizing the fertilizer industry, particularly in regions facing food insecurity.

## Abstract (web summary)

The production of ammonia-based nitrogen fertilizers, key to food production, is highly concentrated and therefore susceptible to price volatility and supply chain disruptions. This study examines the cost-competitiveness of a decentralized ammonia industry with low-carbon ammonia production using small modular technologies, such as electric Haber–Bosch or electrocatalytic reduction.

## Main

Achieving net-zero emissions by 2050 will necessitate transformations across several industries and sectors^1–4^. Notably, agriculture plays a vital role in supporting the global population by supplying essential nutrients such as nitrogen, phosphorus and potassium through fertilizers^5–7^. Among them, nitrogen is the most consumed nutrient in agriculture^8^, and its supply relies on either manure (33%) or industrially synthesized ammonia (67%)^5^. Ammonia-derived nitrogen fertilizers are estimated to produce food that feeds 3.8 billion people or half of the global population^6^. The current worldwide requirement for ammonia in fertilizer production is 132 million metric tons (Mt) per year^9^ (2021). However, projections indicate that this demand is expected to increase to 165 Mt yr^–1^ (ref. ^10^) by 2050. This surge is attributed to factors such as population growth and shifts in dietary patterns, which are predicted to increase^11,12^ food demand by at least 50% by 2050. Beyond the production of nitrogen fertilizers, which cover ~70% of ammonia usage^8^, ammonia is also used in the production of plastics, explosives and synthetic fibres^4,8^. Historically, the ammonia industry has developed around ~500 centralized facilities^13^, which rely on the thermocatalytic Haber–Bosch process fed with natural gas (72%), coal (22%) or heavy fuel oil (3%)^14^. Despite being optimized during the twentieth century, the Haber–Bosch process remains an energy- and carbon-intensive process^15–17^, using 29–47 MJ kg^−1^ NH_3_ of energy and emitting 1.5–3 tCO_2_ t^−1^ NH_3_. The production in centralized facilities requires the additional conversion of ammonia into intermediate molecules, such as urea, ammonium nitrate and nitric acid, which carry 55%, 26% and 13%, respectively, of the current global synthetic nitrogen fertilizers and facilitate the transport of ammonia^5^. While 85% of the emissions from the current centralized facilities are associated with ammonia production, 15% of the emissions are temporarily stored as carbon content in urea molecules and released at the point of fertilizer use^5^. The conventional fertilizer production process, marked by high carbon and energy intensity^14–17^, exposure to supply chain shocks^18^, cost implications arising from long-distance transportation and downstream logistics^19,20^, and the substantial carbon footprint of urea production, emphasizes the need to transition from the current centralized industrial production towards a decentralized configuration.

Under the current centralized configuration, net-zero-emissions fertilizer production can be achieved by upgrading existing production technologies with three measures: (1) utilizing fossil fuels with carbon capture and storage (carbon capture route), (2) employing water electrolysis powered by carbon-free electricity and an external carbon source (carbon usage route) and (3) implementing biochemical processes (bioenergy route)^4,6,21^. In a net-zero ammonia industry based on centralized production plants, carbon dioxide molecules would still be required for the conversion of ammonia into urea for transport purposes^5^. While decarbonizing ammonia production is feasible, it may result in trade-offs in terms of energy, land, water and biomass utilization, potentially exacerbating land and water scarcity issues^6^. In the carbon capture route, current production plants based on steam methane reforming would be retrofitted with carbon capture technology, where carbon dioxide molecules would be permanently stored underground or temporarily captured in urea molecules. Since the carbon embedded in the molecule of urea would be released at the point of use, a carbon compensation method (for example, carbon offsetting and carbon dioxide removal)^22^ would still be required to achieve net-zero-emissions fertilizers with this solution. While carbon capture does not substantially affect the energy resources required to produce urea, from an economic point of view it is a cost-intensive solution^8^. In addition, despite the carbon capture rate achieving 98% of plant emissions^23^, the value drops to 60–85% when accounting for emissions from the upstream natural gas supply chain^24^. Alternatively, net-zero ammonia production can be achieved through water electrolysis (carbon usage route), which, however, requires about 25 times more electricity and land, and 50 times more water compared with conventional production methods^6^. In this case, carbon dioxide required for the conversion of ammonia into urea would be supplied from an external source to the process, such as direct air capture fed with renewable electricity, increasing energy demand. Biochemical processes are a land- and water-intensive solution, requiring three and four orders of magnitude more land and water, respectively, than current production methods^6^. In the bioenergy route, hydrogen and carbon dioxide would be produced from steam methane reforming of bio-methane, with additional carbon capture^24^. However, both electrolytic and biogenic hydrogen are subject to constraints of local renewables and feedstock availability^25,26^. In addition, an infrastructure upgrade would be required to transport hydrogen and carbon dioxide^27^. In some countries, with limited renewable potential and land, the import of low-carbon carriers from regions with high renewable potential would be necessary to maintain centralized production fed with low-carbon energy^28^.

The 2022 energy crisis has underscored the interconnectedness between food and energy systems, with a handful of countries controlling the resources necessary for fertilizer production^6^. Moreover, centralized ammonia production exacerbates transportation costs and carbon emissions, posing additional challenges to providing affordable fertilizers to remote and impoverished regions already struggling with food shortages^29^. By embracing decentralized production, it is possible to mitigate these issues, reduce reliance on imports and ensure a more resilient and equitable distribution of vital agricultural nutrients.

Under a scenario of industrial restructuring towards decentralization, ammonia fertilizers could be produced directly at the point of demand, as a full replacement of centralized plants or complementary to retrofitted centralized plants^30,31^. Decentralized production is particularly relevant for meeting the demand in areas with limited infrastructural connections from production plants, such as sub-Saharan Africa^2^. The direct production of ammonia at the demand point presents the advantage of being independent of derived products, such as urea, required in the case of long-distance transport of ammonia. Decentralized ammonia production at the cropland level requires facilities with an average production capacity lower than 15 t of ammonia per day, compared with the average production capacity of 2,000–3,000 t of ammonia per day in current large centralized facilities^32,33^. The electric version of the traditional Haber–Bosch process replaces the first step of ammonia production based on steam methane reforming with an electrolyser fed with electricity while keeping the second step of ammonia synthesis unaffected. Beyond the electric Haber–Bosch, other technologies, with low operating temperatures and pressures, are suitable for decentralized ammonia production^34^. These technologies include non-thermal plasma-activated nitrogen fixation, photocatalytic nitrogen reduction and direct electrocatalytic nitrogen reduction^34^. Non-thermal plasma-activated nitrogen fixation is based on the activation of nitrogen without a catalyst by generating highly energetic electrons^35^. Photocatalytic nitrogen reduction resembles the artificial photosynthesis of ammonia directly from sunlight, nitrogen and water^36^. Direct electrocatalytic nitrogen reduction (electrocatalysis) can produce ammonia from catalysis based on the direct conversion of water and nitrogen^37,38^.

This study analyses the spatially explicit cost of on-site ammonia production from decentralized technologies while accounting for the spatially explicit local demand for fertilizers that this technology could supply. This approach enables the quantification of the aggregated global fraction of demand that can be cost-competitively satisfied by small-scale decentralized ammonia production technologies, along with the respective accurate locations for optimal deployment. The magnitude of this demand fraction is a measure of the advantage of shifting the current centralized fertilizer production industry to decentralized production. Most techno-economic analyses in process engineering underscore the cost of decentralized ammonia production without considering agricultural demand^34,39^, whereas others focus on techno-environmental factors^30,40^. Simultaneously, food production analyses focus on nitrogen demand (that is, the nutrient that can be released by ammonia use) in agriculture^41,42^, independently from supply routes for synthetic nitrogen. This work bridges the gap between the local cost of deploying technologies for low-carbon ammonia decentralized production, their potential use to supply ammonia demand on croplands and the cost of ammonia production in the current centralized industry. The findings of this work can be instrumental for decision-makers in designing targeted incentives to promote the adoption of decentralized technologies in specific locations. In addition, the results of this study establish a benchmark for industrial players within the fertilizer industry in adopting different technological solutions to transition from carbon-intensive to net-zero ammonia production technologies.

## Results

### Ammonia supply–demand cost-competitiveness

**Fig. 1:**
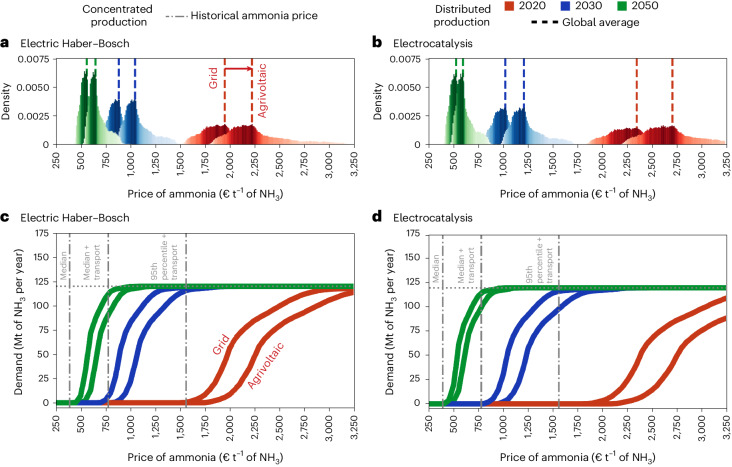
Cumulative ammonia demand cost-competitively supplied with decentralized ammonia production. **a**,**b**, The cost of ammonia for decentralized production is derived from the pixel-level levelized cost of electricity-feeding electric Haber–Bosch (**a**) and electrocatalysis (**b**). The global distribution of the cost of ammonia production is calculated at the pixel level, highlighting the global average value (vertical dashed lines). **c**,**d**, The local cost of decentralized ammonia production is combined with the local demand for ammonia to derive the global fraction of ammonia demand that can be cost-competitively supplied with electric Haber–Bosch (**c**) and electrocatalysis (**d**). For each technology, two systems are considered: connected to the grid with electricity from a mix of conversion technologies and fed with electricity from agrivoltaic solar panels. Reference costs of ammonia production from centralized production are €390 t^−1^ and €780 t^−1^ of NH_3_, chosen from the median and 95th percentile. In addition, the cost of logistics for transporting ammonia is added to the two prices, resulting in a twofold increase in the price of ammonia at the demand point: €780 t^−1^ and €1,560 t^−1^ of NH_3_. The cost-competitiveness of decentralized production varies substantially depending on the cost assumptions for the novel technologies and the reference price of ammonia at the demand point. Source data

To assess the competitiveness of decentralized ammonia production, we combine spatially explicit demand for ammonia as a synthetic nitrogen fertilizer (Fig. 1) with the spatially explicit cost of ammonia from decentralized production technologies. We assume that production technologies are driven either by electricity from the grid, that is, electricity from a mix of power production technologies, or by electricity from an agrivoltaic system, that is, photovoltaic panels installed on agricultural land, which integrate electricity generation and crop production^43–46^. In the case of electricity from the grid, ammonia can be produced with continuous operation, whereas in the case of electricity from agrivoltaics, the operation of the system depends on the local capacity factor of the solar panels and requires additional storage of hydrogen to allow continuous operation of the ammonia synthesis loop. By considering the capital expenditure of the components of the technologies and the variable cost combined with the local levelized cost of electricity, we quantify the local cost of ammonia production based on electric Haber–Bosch and electrocatalysis technologies for ammonia production. In addition, we compare the local cost of ammonia production with the historical ammonia market price to identify the fraction and location of ammonia that can be cost-competitively supplied from decentralized production. Historical data of ammonia market prices are taken from the World Bank commodity price database for 2008–2022^47^ (Supplementary Section 3). While traded commodity prices are influenced by regional spot markets, end-user fertilizer prices are location-specific and contingent on the distance from centralized production plants in the case of land transport or the proximity to terminals in the case of overseas trade^19^. Additional heterogeneity among end-use prices is determined by country-specific taxes and the margins of the companies involved^19^. By considering two extreme scenarios of production and transport costs, we ensure to include the full spectrum of possible fertilizer prices at the demand point. Specifically, we assume fertilizer prices at the demand point to vary between prices of production in the absence of supply shocks with negligible transport cost (that is, median price equalling €390 t^−1^) and prices of production under supply shocks with extreme transport costs (that is, 95th percentile + transport price equalling €1,560 t^−1^) (additional information provided in Supplementary Section 3). Beyond the lower and upper bounds of fertilizer prices mentioned, we consider an intermediate case representative of the fertilizer price in the absence of supply shocks with transport cost (that is, median + transport price equalling €780 t^−1^) and a case representative of the fertilizer price under supply shocks but negligible transport cost (that is, 95th percentile equalling €780 t^−1^). The following text mainly refers to the latter price as the median + transport price.

**Fig. 2:**
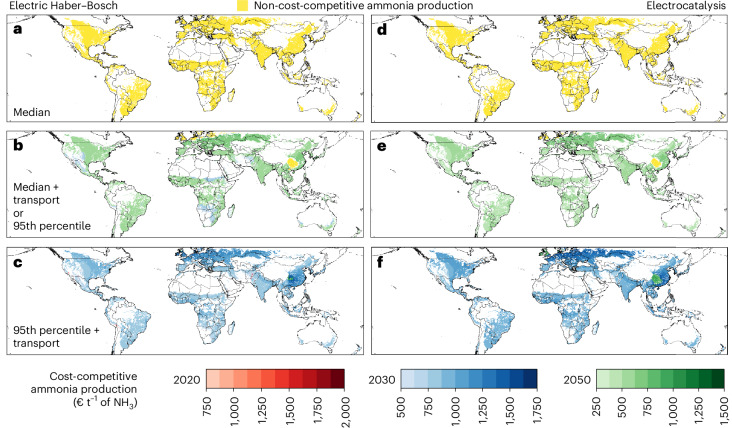
Location of ammonia demand on croplands in the current scenario supplied by either decentralized electric Haber–Bosch or decentralized electrocatalysis in the grid-connected configuration. For each pixel, the cost of ammonia production from decentralized technologies is determined based on the earliest year that achieves cost-competitiveness among 2020 (red), 2030 (blue) and 2050 (green). The cost of decentralized production is compared with the cost of ammonia production from centralized production and the cost of ammonia at the demand point, inclusive of the cost of transport. Reference costs of ammonia production from centralized production are €390 t^−1^, €780 t^−1^ and €1,063 t^−1^ of NH_3_, chosen from the median, 95th percentile, and maximum prices of the historical ammonia market price, respectively. In addition, the cost of logistics for transporting ammonia is added to the three prices, resulting in a twofold increase in the price of ammonia at the demand point. **a**,**d**, Ammonia production by decentralized electric Haber–Bosch (**a**) or decentralized electrocatalysisis (**d**) is never cost-competitive with centralized fossil-based production under low market prices from centralized production and excluding the cost of transport of ammonia. **b**,**c**, Cost-competitiveness based on electric Haber–Bosch is reached for the projected technological development in 2030 and 2050 and in comparison with the median cost of production combined with the cost of transport (equivalent to the 95th percentile cost of ammonia production) (**b**) and the 95th percentile cost of production with the additional cost of transport (**c**). **e**,**f**, Cost-competitiveness based on decentralized electrocatalysisis is reached for the projected technological development in 2030 and 2050 and in comparison with the median cost of production combined with the cost of transport (equivalent to the 95th percentile cost of ammonia production) (**e**) and the 95th percentile cost of production with the additional cost of transport (**f**). Yellow-coloured pixels represent regions where decentralized production is not cost-competitive. Values relative to **a**–**c** are presented in Table 1 clustered at the continental level. The maps are created with the Matplotlib and Geopandas packages for Python^70,71^. Source data

Figure 1 shows the comparison of the cost of ammonia production from decentralized technologies with three different historical prices of ammonia from centralized production plants. Derived from our geospatial analysis, the figure presents the global distribution of ammonia production cost under current (2020, to avoid distortion from the Russian invasion of Ukraine in 2022) and prospective technological developments in 2030 and 2050 for electric Haber–Bosch (Fig. 1a) and electrocatalysis (Fig. 1b). We consider two configurations of ammonia production systems: (1) connected to the grid (grid in Fig. 1) and (2) with electricity supply from agrivoltaic solar panels (agrivoltaics in Fig. 1). The cost of electricity used in the calculations depends on the local solar irradiation at a pixel level, independent of the ammonia production systems. In the case of the agrivoltaic system, the cost of ammonia depends on the local capacity factor, affecting the electrolyser capacity. In addition, the final cost of ammonia production depends on the cost of hydrogen storage to allow continuous operation of the ammonia synthesis loop in the case of an agrivoltaic system^37^. The impact of the capacity factor and the cost of storage is highlighted by the shift in the average ammonia price distribution in 2020, 2030 and 2050 (vertical dashed lines in Fig. 1a,b).

The cumulative demand for ammonia over the cost of decentralized production in Fig. 1 allows us to quantify the demand fraction that could be cost-competitively supplied based on decentralized technologies. The lower technology readiness level of electrocatalysis^34^ (TRL 1–3) implies higher ammonia production costs with respect to the more mature electric Haber–Bosch (TRL 6–7 in the case of a proton-exchange membrane electrolyser and 8–9 in the case of an alkaline electrolyser) in scenarios of short- to medium-term technology deployment (2020, 2030). Under the assumptions of increased technological performance and reduced capital expenditure, electric Haber–Bosch and electrocatalysis present similar production costs per unit of ammonia by 2050. Independent of the technology and its development, electric Haber–Bosch and electrocatalysis are never cost-competitive with the median historical prices of ammonia production from centralized plants in supply chain operations without shocks (median in Fig. 1c,d). When including the cost of transport to the median cost of ammonia production in supply chain operations without shocks, 5% (6 Mt yr^−1^) of ammonia demand can be cost-competitively supplied with decentralized electric Haber–Bosch by 2030, only in the case of a grid-connected system, 94% and 75% by 2050 in the case of grid-connected and agrivoltaic-based systems, respectively (median + transport in Fig. 1c). Grid-connected electrocatalysis can cost-competitively supply up to 96% (115 Mt yr^−1^) by 2050 (median + transport in Fig. 1d). In the case of ammonia production from centralized plants with supply chain shocks (€ 1063 t^−1^ reference price of ammonia), 76% (92 Mt yr^−1^) and 41% (49 Mt yr^−1^) of ammonia can be cost-competitively supplied by 2030 (100% by 2050) with electric Haber–Bosch with a grid-connected system and an agrivoltaic-based system, respectively (Fig. 1c). Electrocatalysis can cost-competitively supply up to 50% (60 Mt yr^−1^) and 5% (6 Mt yr^−1^) by 2030 (€ 1063 t^−1^ reference price of ammonia) in the case of grid-connected and agrivoltaic-based systems, respectively, with the remaining by 2050 (Fig. 1d). In the case where ammonia prices equal the 95th percentile and additional transport cost, 96% (116 Mt yr^−1^) and 100% (120 Mt yr^−1^) of the demand can be cost-competitively supplied based on electric Haber–Bosch by 2030 (95th percentile + transport in Fig. 1c) in the case of an agrivoltaic system and a grid connected system, respectively, while 82% (98 Mt yr^−1^) and 97% (117 Mt yr^−1^) can be cost-competitively supplied based on electrocatalysis (95th percentile + transport in Fig. 1d). The case of production costs under supply chain shocks with the additional cost of transport (€ 2125 t^−1^ reference price of ammonia) determines the largest price of ammonia at the demand point. Under these conditions, 62% (74 Mt yr^−1^) and 20% (23 Mt yr^−1^) of the global demand for ammonia could be supplied by electric Haber–Bosch already under the current (2020) production costs, by relying on a grid-connected or an agrivoltaic system, respectively (Fig. 1c). The currently low efficiency of electrocatalysis (2020) limits its potential to 10% of the global demand based on a grid-connected system, even under this extreme case of ammonia price (€ 2125 t^−1^ reference price of ammonia) at the demand point (Fig. 1d).

### Spatially explicit decentralized supply cost-competitiveness

On the basis of a spatially explicit demand for ammonia and cost of production from small-scale electric Haber–Bosch and electrocatalysis, we identify regions worldwide where decentralized ammonia production can be cost-competitive with historical ammonia production from centralized industrial plants. Figure 2 shows the geographical distribution of the fraction of ammonia demand in the earliest year that achieves cost-competitiveness due to technological development for electric Haber–Bosch and electrocatalysis. When the production cost of ammonia is compared with the median of the historical price of centralized ammonia production (€390 t^−1^ of NH_3_), decentralized ammonia supply does not reach cost-competitiveness anywhere in the world (Fig. 2a,d). By contrast, when the local cost of ammonia production is compared with the 95th percentile of the historical price of centralized ammonia production, equivalent to the historical median with the additional ammonia transport cost (€780 t^−1^ of NH_3_, Fig. 2b,e), cost-competitiveness is reached in small regions in South Africa and North America by 2030, in the case of production from electric Haber–Bosch. In the rest of the world, except for Northern Europe and inland China, cost-competitiveness is only reached by 2050 (Fig. 2b,e). When the production cost of ammonia is compared with the 95th percentile of the historical price of centralized ammonia production with the additional cost of transport (€1,560 t^−1^ of NH_3_), cost-competitiveness is reached by 2030 between 96% and 100% of croplands worldwide, depending on the technology and system considered. In this cost comparison, regions with the highest cost of ammonia production based on electrocatalysis reach cost-competitiveness only by 2050. Significative values shown in Fig. 2a–c at the continent level are summarized in Table 1.

**Table 1:** Continent-specific demand and cost-competitiveness based on electric Haber–Bosch with electricity from the grid

Current demand (Mt yr^−1^)	Reference cost traditional Haber–Bosch	Cost-effective demand (% of current demand)		
		2020	2030	2050
Asia 68 Mt yr^−1^ demand	median	**–**	**–**	**–**
	95th percentile or median + transport	**–**	1%	93%
	95th percentile + transport	–	99%	100%
Europe 18 Mt yr^−1^ demand	median	–	–	–
	95th percentile or median + transport	–	–	89%
	95th percentile + transport	–	100%	100%
Africa 5 Mt yr^−1^ demand	median	–	–	–
	95th percentile or median + transport	–	40%	100%
	95th percentile + transport	–	100%	100%
South America 9 Mt yr^−1^ demand	median	–	–	–
	95th percentile or median + transport	–	–	100%
	95th percentile + transport	–	100%	100%
Oceania 2 Mt yr^−1^ demand	median	–	–	–
	95th percentile or median + transport	–	50%	100%
	95th percentile + transport	–	100%	100%
North America 18 Mt yr^−1^ demand	median	–	–	–
	95th percentile or median + transport	–	11%	100%
	95th percentile + transport	–	100%	100%
Global 120 Mt yr^−1^ demand	median	–	–	–
	95th percentile or median + transport	–	5%	94%
	95th percentile + transport	–	100%	100%

The table presents a breakdown of the current demand for ammonia (Mt yr^−1^), which totals 120 Mt yr^−1^ globally, divided by continent (first column). The demand for each continent is segmented based on the percentage that can be met cost-competitively in 2020, 2030 and 2050. Reference costs of ammonia production from centralized plants are €390 t^−1^ and €780 t^−1^ of NH_3_, chosen from the median and 95th percentile, respectively. In addition, the cost of logistics for transporting ammonia is added to the two prices, resulting in a twofold increase in the price of ammonia at the demand point: €780 t^−1^ and €1,560 t^−1^ of NH_3_.

### Continent- and country-specific decentralized supply

By aggregating spatially explicit data from pixels to continent and country levels, we can identify the continent- and country-specific fraction of ammonia demand that can be cost-competitively supplied with decentralized production based on electric Haber–Bosch or electrocatalysis. In addition, this analysis identifies the continent- and country-specific fraction of ammonia for which centralized production is expected to be the most competitive option, independent of future developments in technologies for decentralized production.

Table 1 presents the fraction of ammonia demand that small-scale decentralized electric Haber–Bosch can cost-competitively supply at the continent level. The largest fraction is in Africa where decentralized production can supply up to 40% (2 Mt yr^−1^) of the continental demand (5 Mt yr^−1^), in the case of production and transport cost of ammonia summing up to the 95th percentile of the historical price.

**Fig. 3:**
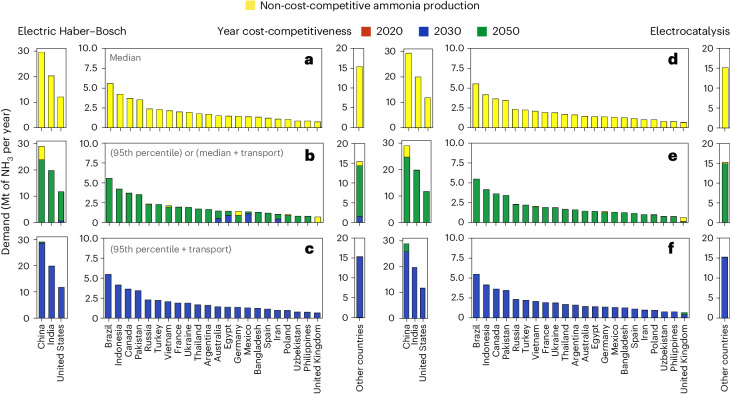
Aggregated demand for ammonia at the country level for the top 25 countries for ammonia demand in the grid-connected configuration. **a**–**f**, Comparison of ammonia production is based on technology cost in 2020 (red) and assumptions of technological development for 2030 (blue) and 2050 (green) for electric Haber–Bosch (**a**–**c**) and electrocatalysis (**d**–**f**). Reference costs of ammonia production from centralized plants are €390 t^−1^, €780 t^−1^ and €1,560 t^−1^ of NH_3_, chosen from the median (**a**,**d**), the 95th percentile (**b**,**e**), and the 95th percentile cost of production with the additional cost of transport (**c**,**f**), respectively. Yellow-coloured stacks represent the demand for which decentralized production is not cost-competitive compared with centralized production.

Country-level data are shown in Fig. 3, showcasing the proportion of demand that cannot be met cost-competitively through decentralized production (yellow), along with the respective years when cost-competitiveness is achieved (red for 2020, blue for 2030 and green for 2050). Figure 3b,e shows that decentralized production can be cost-competitive with the median of the price when the transport cost to the demand point is accounted for in the comparison, or with the 95th percentile of the production cost alone, with the technological development achieved by 2050. However, even in these cases, technologies for decentralized ammonia production are never cost-competitive for 17% of demand in China, 36% in Germany and 100% in the United Kingdom at the country level with electric Haber–Bosch (Fig. 3b). Trends for electrocatalysis are similar; however, the technological development projected for 2050 is required to make electrocatalysts an alternative option to centralized ammonia production (Fig. 3e).

## Discussion

Across the world, fertilizer use is heterogeneous, with regions characterized by nitrogen shortages that limit food production and regions where excessive use of nitrogen harms biodiversity, water quality and human health, and generates greenhouse gas emissions^48^. The need to transport ammonia from centralized production plants to decentralized final points of use in croplands has led to the conversion of volatile and toxic ammonia into stable chemical products, such as urea, ammonium nitrate and nitric acid, currently representing 91% of nitrogen fertilizer use^5^. While about 90% of CO_2_ emissions in nitrogen fertilizer production derive from the synthesis of ammonia^8^, additional CO_2_ emissions come from the use of urea (CH_4_N_2_O), produced^14^ from the synthesis of ammonia and CO_2_. At the point of use, by reacting with water, ammonia or urea delivers reactive nitrogen as a plant nutrient, with urea additionally releasing CO_2_ into the atmosphere. Currently, ~480 Mt of carbon dioxide equivalent (CO_2_e) per year is emitted from the production of nitrogen fertilizers, in addition to ~90 MtCO_2_e yr^−1^ embedded in urea molecules, later released into the atmosphere at the point of use of the fertilizer^5^. Although most nitrogen losses occur at the demand point, with unintentional environmental consequences such as algae bloom^35^, the long-distance transport required with centralized production of ammonia increases the risk of nitrogen losses along the supply chain between production and demand points compared with on-site productions^49,50^.

Decentralized ammonia production has the double advantage of not requiring conversion into an intermediate carrier such as urea, relying on the supply of carbon dioxide for synthesis. Instead of urea, commercialized in the form of solid prills and diluted for distribution on croplands, ammonia fertilizers can be produced on-site in the form of anhydrous or aqueous ammonia. Anhydrous ammonia allows to reach high ammonia concentrations but requires high pressures for conservation in gaseous form and sophisticated machines for injection into the soil^19,31^. Aqueous ammonia contains lower ammonia concentrations but can be stored under ambient conditions in liquid form and can rely on existing irrigation systems for distribution—a technique known as fertigation^19,31^—or agricultural sprayers, such as backpack or boom sprayers.

In the case of agrivoltaic systems, relying on the coupling of electricity production from solar panels with technologies for ammonia production, the cost of ammonia at the demand point is stabilized and independent of fluctuations in the price of fossil fuels and geopolitical events. However, this configuration determines a dependence on the capacity factor of the solar panel, leading to a fivefold increase in fixed costs, reflected in a higher unit cost of ammonia production. In the case of electric Haber–Bosch, the system configuration based on agrivoltaic requires an additional capacity for hydrogen storage to guarantee the continuous operation of the ammonia synthesis loop, increasing the fixed costs of the technology by more than 20% (see agrivoltaic systems in Supplementary Section 2). The ammonia synthesis loop involves temperature- and pressure-dependent chemical reactions. Continuous operation minimizes energy requirements by maintaining design conditions that optimize catalytic activity and the chemical reaction rate for ammonia production^51^. The largest fraction of fixed and variable costs is associated with the capital expenditure of the electrolyser and the electrocatalyst. The connection of the system to the grid avoids the impact of intermittent electricity production from renewables. In this case, the final cost of ammonia and the carbon intensity of the electricity used depend on the power market and the country-specific mix of conversion technologies. To keep the analysis independent from country-specific considerations and predictions of the price of electricity in 2030 and 2050, we based our analysis on the levelized cost of electricity derived from spatially explicit solar irradiation. An additional advantage derived from decentralized fertilizer production is the minimal requirement of storage for fertilizers. While in the case of centralized production the delivery of ammonia based on trade (a couple of times per year) requires storage vessels to accommodate agricultural consumption patterns, decentralized production allows continuous production and application^29^.

The comparison of centralized and decentralized ammonia production within this analysis is based on historical ammonia market prices^47^ between 2008 and 2022 and the cost of ammonia production at the demand points. The accurate quantification of the price of ammonia for the final user from centralized production would include the additional cost of transportation and distribution from the production plants to the final demand points. Transportation costs for fertilizers are country-specific, ranging from 12% in Thailand to 39% in Mozambique of the fertilizer price at the point of consumption^19^. Within sub-Saharan Africa, the most expensive local market price data were recorded in the landlocked countries of Burundi, Uganda and Burkina Faso, reaching up to €1,307 t^−1^ of urea^20^. For a more precise assessment of decentralized ammonia production’s competitiveness, it is essential to consider technology- and country-specific incentives for climate action, such as the United States’ Inflation Reduction Act^52^ and the European Union’s Net Zero Industry Act^53^. The results of this analysis depend on assumptions made about the costs associated with the technologies, derived from relevant literature. These results are sensitive to variations in certain key parameters. Specifically, the discount rate, capital expenditure in the levelized cost of electricity, and the efficiencies of the electrolyser and electrocatalyst are the main factors influencing the median value of the global distribution of ammonia production (see Supplementary Section 4).

Achieving a net-zero-emissions scenario based on centralized ammonia production is possible by upgrading existing production plants with carbon capture technologies or with water electrolysis for hydrogen production from renewable electricity and carbon from an external source for urea production. In the former case, the cost of ammonia production is still dependent on the cost of natural gas. In the latter, the main cost of feedstocks is the local cost of electricity from the grid or produced with dedicated renewable technologies. Capturing carbon emissions from ammonia production based on natural gas implies an additional cost of €85–130 t^−1^ of NH_3_, under the assumption of a 95% plant-level capture rate and a transport cost^14^ of €15–35 t^−1^ of CO_2_. These additional costs reduce approximately to €35–70 t^−1^ of NH_3_ in the case of carbon capture based on autothermal reforming, which also allows a 98% plant capture rate^14^. Estimated differential costs in ammonia production between centralized traditional Haber–Bosch and centralized water electrolysis plants vary^8^ approximately between −€70 t^−1^ and +€140 t^−1^ of NH_3_, depending on the cost of natural gas (€6 MWh^−1^ and €27 MWh^−1^, respectively), for an electricity cost of €25 MWh^−1^, which allows reaching a breakeven point between the two technologies at a natural gas cost of approximately €13 MWh^−1^. Cost differences also vary^8^ between −€85 t^−1^ and €460 t^−1^, depending on the cost of electricity (€20 MWh^−1^ and €77 MWh^−1^, respectively), for a natural gas cost of €27 MWh^−1^, which allows reaching a breakeven point between the two technologies at an electricity cost of €29 MWh^−1^. Ammonia production from water electrolysis becomes cost-competitive^8^ with the carbon capture route for electricity costs lower than the breakeven points of €14 MWh^−1^ and €34 MWh^−1^, for natural gas costs of €6 MWh^−1^ and €27 MWh^−1^, respectively. These estimates exclude the cost of CO_2_ involved in the conversion of ammonia into urea. The carbon intensity of ammonia in the carbon usage route depends on the carbon intensity of the electricity-feeding technology. Reference values are 458 kg of CO_2_e per MWh of electricity as the world average carbon intensity^54^ in the case of grid-connected systems, reducing to 87.5 kg CO_2_e per MWh in the case of electricity from solar photovoltaic panels (range 23–183 kg CO_2_e per MWh)^55^. The total electricity required to supply the assumed 120 Mt yr^−1^ of ammonia demand based on decentralized technologies is estimated to be less than 10% of the total global electricity consumption recorded in 2019, which amounted^56^ to 22.8 PWh yr^−1^. Local analyses of the grid are required to assess the impact of these technologies on safety and reliability.

The restructuring of the ammonia production industry towards electrified and decentralized production would affect the entire fertilizer supply chain, which currently depends on energy imports and trade of final products. In fact, in the current centralized fossil-based ammonia production, 8% of global food demand relies on ammonia produced from imported natural gas, while an additional 12% directly relies on ammonia import^6^. Therefore, food crop prices depend on fertilizer prices subject to the highly volatile and uncertain market of fossil fuel feedstocks, mostly natural gas^35^, and the additional cost of transportation to final demand points^31^. Limited access to fertilizers and an increase in the marginal cost of crops can favour undernourishment and food insecurity^29^. While centralized production plants face the risk of becoming stranded assets because of the capital intensiveness of their construction and additional investments required for their retrofitting, the widespread use of small-scale modular technologies can occur in a short time due to the maturity of electric Haber–Bosch and the potential short-term (by 2030) technological developments such as electrocatalysis. In addition, these technologies require minimal upgrades in infrastructure, contrary to large-scale centralized ammonia production plants.

## Methods

### Ammonia demand

We consider global demand for ammonia from a spatially explicit database^42^ providing nitrogen use per crop and per fertilizer type in 2020. To our knowledge, this database is the most up-to-date source of N fertilization data and representative of a total demand for ammonia of 120 Mt yr^−1^, which is the closest value to the 132 Mt yr^−1^ total global demand for ammonia for agricultural use derived from estimations by the Food and Agriculture Organization^9^ in 2021. The use of spatially explicit data of demand in this study is necessary for combining with the location-specific cost of ammonia production from distributed technologies, derived from the spatially explicit levelized cost of electricity (that is, the local cost of renewable electricity production dependent on location-specific solar irradiation and capacity factor). We consider the fertilizer nitrogen demand for 18 major crops, vegetables, fruits and other crops (see Supplementary Table 1 in Supplementary Section 1) and derive the corresponding amount of ammonia assuming a stoichiometric ratio of 1.21 kg of ammonia per kg of nitrogen. In estimating the demand for nitrogen, we include nitrogen from different types of synthetic fertilizer, excluding nitrogen from manure and crop residues. The original data have a resolution of 0.083° (10 km × 10 km at the Equator), which were aggregated at global, continental and country scales. At a pixel level, the maximum local demand for ammonia over harvested areas (discretized in pixels from 10 km^2^ to 175 km^2^ in size) is lower than 71 t d^−1^ (equivalent to 26 kt yr^−1^). The mass unit of measure used in article refers to metric tons (t).

### Energy production

We consider electric Haber–Bosch and electrocatalysis as candidate technologies that could be deployed for distributed ammonia production. Low-carbon production of ammonia requires any of these technologies to be fed with renewable electricity, assumed here to be provided either from the grid or from small-scale solar panels. In the latter case, electricity is assumed to be supplied by an agrivoltaic system. This typology of system integrates crop production with power generation from photovoltaics. Photovoltaic panels are installed on the ground with sufficient space to allow the use of farming equipment and operations. Agrivoltaic systems are chosen due to their dual advantage of producing renewable electricity while providing shade for crops, limiting the evaporation of water from the soil and leading to 4–29% water saving^44–46^. The yearly average solar power production, *S*, is a technology-, time-, space- and weather-dependent parameter. We quantify this parameter with a bottom-up approach, based on a yearly average geographical discretization at 0.75° × 0.75° grid resolution (about 80 km × 80 km at the Equator), resampled to 0.083° × 0.083° grid resolution (about 10 km × 10 km at the Equator). The energy production from solar photovoltaics for all cells, *G*, is computed as:

1 $${{{S}}}_{{{i}}}^{\;{\mathrm{solar}}}={\eta }^{\,{\mathrm{solar}}}{{{I}}}_{{{i}}}\qquad\quad\forall {{i}}\in \,{{G}}$$

where ${S}_{i}^{\;{\mathrm{solar}}}$ (TWh km^−2^ yr^−1^) is the yearly energy production from solar panels per square kilometre in grid cell *i*, *η*^solar^ is the conversion efficiency of solar panels^45^ and *I*_*i*_ (TWh km^−2^ yr^−1^) is the yearly average global horizontal irradiation^57^ in cell *i*.

### Electricity and ammonia production cost

Three technologies can be assumed as representative of ammonia production evolution^16^. The traditional Haber–Bosch process for centralized ammonia production is taken as a reference in this study based on historical data of ammonia market price (Supplementary Section 3). Given its high readiness level, the small-scale electrified Haber–Bosch process for distributed ammonia production can be considered a second-generation technology^16,58^. Finally, due to its low technological development, the direct conversion of nitrogen and water for distributed ammonia production can be considered a third-generation technology^16,34^. Here we derive the production cost of ammonia from the second- and third-generation technologies for distributed ammonia production, namely electric Haber–Bosch and direct electrochemical reduction. In the electric Haber–Bosch process, we assume hydrogen production from a proton-exchange membrane electrolyser, which is a commercially available technology and presents load flexibility advantages compared with other electrolyser types^59,60^.

To produce low-carbon ammonia, both technologies have to be powered with renewable electricity. Here we assume electricity from the grid or generated from solar photovoltaics in an agrivoltaic system installed on croplands. The cost of ammonia production per technology is computed in two steps. First, we compute the levelized cost of electricity independently of the technology for ammonia production under cost assumptions for 2020, 2030 and 2050. Then, we derive the final cost of ammonia based on the energy consumption, capital and variable cost of each technology. Since our analysis is based on the combination of spatially explicit cost of ammonia production with spatially explicit demand for ammonia, we assume the levelized cost of electricity as the variable cost for both grid-connected and photovoltaics-based systems. In the following, we summarize the steps for the derivation of the marginal cost of ammonia production from decentralized technologies. Further details are provided in Supplementary Section 2.

We compute the cost of energy feeding the technologies for ammonia production based on the levelized cost of electricity, ${{{c}}}_{{{i}}}^{{{\mathrm{e}}}}$ (€ kWh^−1^), according to ref. ^61^, in every cell *i*:

2 $${{{c}}}_{{{i}}}^{{\mathrm{e}}}=\left({{C}}+\mathop{\sum }\limits_{{{t}}=1}^{{{T}}}{({O}}/{(1+{{r}})}^{{{t}}})\right)/\left(\mathop{\sum }\limits_{t=1}^{T}\left({{{S}}}_{{{i}}}/{{{{F}}}_{{{i}}}}{(1-{{d}})}^{{{t}}}/{(1+{{r}})}^{{{t}}}\right)\right)\,\qquad\forall {{i}}\in {{G}}$$

where *C* (€ kW^−1^) is the capital expenditure for solar panels, *O* (€ kW^−1^ yr^−1^) is operation and maintenance fixed costs, *r* is the discount rate, *d* is the degradation rate, *T* (yr) is the lifetime of solar panels, *S*_*i*_ (kWh yr^−1^) is the solar energy production derived from equation (1) and *F*_*i*_ (kW) is the capacity installed according to equation (3). The installed capacity, *F*_*i*_ (kW), is computed as:

3 $${{{F}}}_{{{i}}}={{{S}}}_{{{i}}}/{{{f}}}_{{\rm{i}}}/8,760\,\qquad\quad\forall {{i}}\in {{G}}$$

where *f*_*i*_ is the capacity factor^57^ in cell *i*.

The cost of producing ammonia from electricity from solar panels feeding the electric Haber–Bosch and the direct electrochemical reduction is determined in this study using a reference methodology^62^. For both technologies, the marginal cost of ammonia is computed as:

4 $${{{c}}}_{{{i}}}^{\,{{a}}}={{{v}}}_{{{i}}}+{{{w}}}_{{{i}}}\,\qquad\quad\forall {{i}}\in {{G}}$$

where ${{{c}}}_{{{i}}}^{\,{{a}}}$ (€ t^−1^ of NH_3_) is the total cost of ammonia production in cell *i*, *v*_*i*_ (€ t^−1^ of NH_3_) is the variable cost due to the production of ammonia in cell *i* and *w*_*i*_ (€ t^−1^ of NH_3_) is the fixed cost of the system in cell *i*.

In the case of electric Haber–Bosch, the variable costs include the electricity feeding the electrolyser for hydrogen production, the ammonia synthesis loop and the air separation unit (ASU) for nitrogen production from air:

5 $${{{v}}}_{{{i}}}={{{c}}}_{{{i}}}^{\,{{e}}}\times \left({{{e}}}_{{\mathrm{eletrolyser}}}/{{{m}}}_{{\mathrm{reaction}}}/{\eta }_{{\mathrm{electrolyser}}}+{{{e}}}_{{\mathrm{NH}}3{\rm{synthesis}}}+{{{e}}}_{{\rm{ASU}}}\right)\,\qquad\quad\forall {{i}}\in {{G}}$$

where ${{{c}}}_{{{i}}}^{\,{{e}}}$ (€ MWh^−1^) is the levelized cost from photovoltaic in cell *i*, *e*_eletrolyser_ (MWh t^−1^ of H_2_) is the lower heating value of hydrogen, *ƞ*_electrolyser_ is the electrolyser efficiency with respect to the lower heating value of hydrogen, *e*_NH3synthesis_ (MWh t^−1^ of NH_3_) is the energy consumption for ammonia synthesis from hydrogen feedstock and *e*_ASU_ (MWh t^−1^ of NH_3_) is the energy consumption for the operation of the ASU.

In the case of electrocatalysis, the variable cost is composed of the energy required in the (electro)catalytic system to supply the ASU:

6 $${{{v}}}_{{{i}}}=\left({{e}}/\eta +{{{e}}}_{{\mathrm{ASU}}}\right){\,{{c}}}_{{{i}}}^{{{e}}}\,\qquad\quad\forall {{i}}\in {{G}}$$

where ${{{c}}}_{{{i}}}^{\,{{e}}}$ (€ MWh^−1^) is the levelized cost of electricity from photovoltaic in cell *i*, *e* (MWh t^−1^ of NH_3_) is the enthalpy of reaction of nitrogen electroreduction to ammonia, *e*_ASU_ (MWh t^−1^ of NH_3_) is the energy consumption for the operation of the ASU and *ƞ* is the conversion efficiency of electrocatalysis.

While the variable cost is a cell-specific quantity, due to the dependence on the levelized cost of electricity in cell *i*, the fixed cost can be dependent on the local capacity factor, in the case of electricity from an agrivoltaic system, or independent of spatial resolution, in the case of electricity supplied from the grid. For electric Haber–Bosch, the fixed cost is derived from the electrolyser, hydrogen storage capacity, the Haber–Bosch synthesis loop, the ASU and the cost of maintenance of the plant:

7 $${{{w}}}_{{{i}}}={{{c}}}_{{\rm{electrolyser}},{{i}}}+{{{c}}}_{{\rm{NH}}3{\rm{synthesis}}}+{{{c}}}_{{\rm{OaM}}}+{{{c}}}_{{\rm{ASU}}}+{{s}}\,\qquad\quad\forall {{i}}\in {{G}}$$

where *c*_electrolyser,*i*_ (€ t^−1^ of NH_3_) is the capex of the electrolyser per unit of ammonia produced in cell *i*, *c*_NH3synthesis_ (€ t^−1^ of NH_3_) is the fixed cost of ammonia synthesis, *c*_OaM_ (€ t^−1^ of NH_3_) is the fixed cost for operation and maintenance, *c*_ASU_ (€ t^−1^ of NH_3_) is the capex of the ASU, *s* (€ t^−1^ of NH_3_).

In the case of electricity supplied from the grid, the cost of storage, *s*, is assumed equal to zero, and the capacity factor, affecting the capex of the electrolyser, is assumed equal to the global average for solar panels in every cell *i*.

For electrocatalysis, the fixed cost is derived from two components of the system, electrocatalyst and ASU, in addition to the cost of maintenance of the plant:

8 $${{{w}}}_{{{i}}}={{{c}}}_{{\rm{catalyst}},{{i}}}+{{{c}}}_{{\rm{OaM}}}+{{{c}}}_{{\rm{ASU}}}\,\qquad\quad\forall {{i}}\in {{G}}$$

where *c*_OaM_ (€ t^−1^ of NH_3_) is the fixed cost for operation and maintenance, *c*_ASU_ (€ t^−1^ of NH_3_) is the capex of the ASU and *c*_catalyst,*i*_ (€ t^−1^ of NH_3_) is the capex of catalyst per unit of ammonia produced in cell *i*.

In the case of electricity supplied from the grid, the capacity factor affecting the capex of the catalyst is assumed to be equal to the global average solar panel capacity factor in every cell *i*.

### Caveats

With the advent of electrification, the necessity for extensive centralized production could diminish, thereby paving the path for electrified decentralized production^30^. Currently, the need for a limited number of centralized production plants lies in the large capital investments required to sustain the high temperatures (>1,000 K for hydrogen production from natural gas) and pressures (>250 bar for ammonia synthesis) of the traditional thermal Haber–Bosch process^15,17^. In the traditional Haber–Bosch process, these investments are mitigated by a shift from decentralized to centralized production with up to 50% reduction in the final cost of ammonia^63^. Instead, in processes requiring lower temperatures and pressures, a shift to centralized production would only lead to limited reductions in the final cost of ammonia (<15% in the electric Haber–Bosch process)^63^.

If the production of ammonia-derived fertilizers were to maintain the same current carbon intensity^5^ (~3.7 MtCO_2_e Mt^−1^ NH_3_) and the same proportion of urea manufacturing^5^ (0.9 Mt CH_4_N_2_O Mt^−1^ NH_3_) with the demand of ammonia^10^ in 2050 (165 Mt yr^−1^), carbon emissions for the production of ammonia in the fertilizer industry would reach 734 MtCO_2_e yr^−1^, with an additional 108 MtCO_2_e yr^−1^ within urea molecules. Instead, if by 2050 all ammonia were produced in a decentralized configuration from renewable electricity (that is, electrocatalysis or electrified Haber–Bosch fed with renewable electricity) with a carbon intensity^15^ of 0.05 tCO_2_ t^−1^ NH_3_ and without the need for producing urea, emissions for ammonia production in the fertilizer industry would only be approximately 8 MtCO_2_e yr^−1^. The complete shift to decentralized ammonia production would lead to savings of 834 MtCO_2_e yr^−1^.

While our analysis focuses on providing a comparison of centralized and decentralized ammonia production costs based on current information, different factors affect the future price of ammonia in centralized and decentralized configurations. The international trade of ammonia as a commodity, required in the case of production from a centralized configuration, implies that future ammonia prices will be determined by the law of supply and demand. Depending on the future routes of centralized ammonia production, ammonia price will depend on either the price of natural gas for carbon capture routes from the commodity market or the price of electricity for electrolytic hydrogen production in the carbon usage route from the power market. While 70% of current global demand for ammonia is associated with fertilizers^8^, future demand is predicted to involve the use of ammonia as a fuel in shipping and power production sectors^64,65^, in addition to the current uses. The average increase in ammonia demand until 2050 varies between 2.8% and 3.7% per year^8^, depending on future demand scenarios. Supply of ammonia can grow at similar rates, thus having a limited impact on market price. However, the technology upgrade for centralized ammonia production could have a long-term impact on the cost of ammonia production. In the context of decentralized technologies for ammonia production, there is no commodity market involved, as suppliers and consumers of ammonia are the same. In this case, the price of ammonia equals the cost of ammonia production and is highly dependent on the development and commercialization of small-scale electric Haber–Bosch and electrocatalysis.

The electrical synthesis of ammonia, based on electric Haber–Bosch or electrocatalysis, is among the solutions that can be implemented at a farm level to achieve net-zero emissions in agriculture while maintaining high crop productivity^66^. Although this analysis is limited to the deployment of electric Haber–Bosch and electrocatalysis, other technologies exist for distributed ammonia production operating at low temperatures and atmospheric pressure^2^. Non-thermal plasma-activated nitrogen fixation allows the production of ammonia with theoretical energy consumption lower than the Haber–Bosch process. However, plasma processes are still in the research stage as a technological advancement aimed at reducing energy requirements^35^. Photocatalytic nitrogen reduction can be fed directly with solar energy. However, its commercial use requires a more advanced understanding of the effect of reaction mechanisms, solution compositions and material activity on performance. In addition, current experimental research lacks accurate measurement standards, limiting the reproducibility of results^36^. Beyond technological solutions, biological nitrogen fixation, based on ammonia production from bacteria, is the oldest technique used by farmers to increase the level of nitrogen as a plant nutrient^67^. The main bacteria for nitrogen fixation are present in the roots of legumes, but other nitrogen-fixing bacteria can be found in alder trees and aquatic ferns^67^. While currently only legumes such as beans and peas can host nitrogen-fixing bacteria, genetic engineering could lead to biological nitrogen fixation in cereal crops^68^. A major source of nitrogen, not considered in this study, is manure, which supplies approximately 55 Mt yr^−1^ of nitrogen globally, equivalent to 33% of the total global synthetic nitrogen fertilizers^5^.

The discussion surrounding distributed ammonia production naturally leads to enquiries regarding the ease of converting ammonia into potentially hazardous substances, such as ammonium nitrate (NH_4_NO_3_), and the corresponding risk management strategies. However, ammonia is a chemical already largely produced without creating national security issues. Nonetheless, the production of ammonia in less controlled environments might lead to potential misuse for munition production. Although the scope of this work does not cover the specific aspects of security risks, recognizing its significance highlights the need for future research to explore this aspect.

Political measures aimed at curbing emissions from the ammonia industry could increase the cost of ammonia. Based on the findings of our extensive global analysis, which underscores the potential for a reconfiguration of the global ammonia industry through decentralized production, a reduction in the supply cost of ammonia could be achieved in regions where transportation from centralized production represents the largest fraction in the breakdown of fertilizer price. Future research should concentrate on smaller geographical scales and conduct a cost comparison based on existing centralized production plants and the cost of transportation to distribute ammonia demand on croplands. An extensive cost-competitiveness analysis should be carried out considering infrastructural and technological requirements for retrofitting solutions in existing centralized ammonia production plants and country-specific political incentives for the adoption of low-carbon technologies.

### Reporting summary

Further information on research design is available in the Nature Portfolio Reporting Summary linked to this article.

### Supplementary information

Supplementary Information Supplementary Tables 1–5, Figs. 1–6 and Methods 1–4.

Reporting Summary

### Source data

Source Data Figs. 1 and 2 Data represented in Fig. 1 and data required for representation of Fig. 2 with no spatially explicit resolution.

Source Data Fig. 2 Data with spatially explicit resolution (.tiff files), which are combined for representation of Fig. 2.

## Data Availability

The processed data are available at 10.5281/zenodo.8155141 (ref. ^69^). This dataset includes information on the country-specific demand for ammonia and cost-competitiveness of decentralized ammonia production. Source data are provided with this paper.

## Code availability

The complete Python codes used for the calculation and visualization of the results can be accessed via Zenodo at 10.5281/zenodo.8155141 (ref. ^69^). A description of the content of the repository is provided online.
