# Supplementary material for: Cost-competitive decentralized ammonia fertilizer production can increase food security
Source: Nat Food. 2024 May 16;5(6):469–79. doi: 10.1038/s43016-024-00979-y (PMC11199140; doi:10.1038/s43016-024-00979-y)
Supplement: Supplementary file 1 — Supplementary Tables 1–5, Figs. 1–6 and Methods 1–4. [file 43016_2024_979_MOESM1_ESM.pdf]

nature food

Article https://doi.org/10.1038/s43016-024-00979-y

Cost-competitive decentralized ammonia fertilizer production can increase food security

In the format provided by the authors and unedited

Table of contents

S.1 Ammonia demand **2**

S.2 Spatially-explicit cost of ammonia **4**

Levelized cost of electricity 5
Electrocatalysis 7
Electric Haber-Bosch.......... 11
Spatially-explicit cost of ammonia 14
Cost breakdown.......... 15

S.3 Ammonia market price **16**

S.4 Sensitivity analysis.......... **18**

SUPPLEMENTARY REFERENCES **19**

S.1 Ammonia demand

We consider global demand for ammonia[1] from 2020. To the authors' knowledge, this database is the most up-to-date source of N fertilization data, and it is representative of a total demand for ammonia of 120 Mt/y, which is the closest value to the 132 Mt/y total global demand for ammonia for agricultural use derived from estimations by FAO[2] in 2021. We computed the fertilizer nitrogen demand based on the eighteen major crops, plus vegetables, and fruits listed in **Table S1**, and derived the corresponding amount of ammonia assuming stoichiometric 1.21 kg of ammonia per kg of nitrogen.

Table S1. Crops, vegetables and fruits considered for the estimation of the global ammonia demand. The reported value of ammonia demand refer to the aggregated value from the crops, vegetables and fruits in the table[1].

	**2020 demand scenario**
**global ammonia demand**	120.1 Mt/year
**harvested area**	1522.2 million hectares
**fertilizer types**	anhydrous ammonia (AA), ammonium nitrate (AN), ammonium sulphate, (AS), calcium ammonium nitrate (CAN), nitrogen solutions (NS), other N straight (ONS), urea, ammonium phosphate (AP), N K compounds (NK), N P K compounds (NPK), other NP (ONP)
**major crops**	rice, maize, wheat, barley, cereals, sorghum, millet, rye, sugarbeet, crops, sugarcane, palm, sunflower, rapeseed, groundnut, potato, sweetpotato, cassava, cotton, soybean
**vegetables**	artichoke, asparagus, cabbage, carrot, cauliflower, chilli, cucumber, eggplant, garlic, green bean, green broad bean, green corn, green onion, green pea, lettuce, melon, mushroom, okra, onion, pumpkin, spinach, string bean, tomato, vegetable, watermelon
**fruits**	apple, apricot, avocado, banana, berry, blueberry, carob, cashewapple, cherry, citrus, cranberry, currant, date, fig, fruit, gooseberry, grape, grapefruit, kiwi, lemon lime, mango, orange, papaya, peach, pear, persimmon, pineapple, plantain, plum, quince, raspberry, sour cherry, stone fruit, strawberry, tang, tropical
**other crops**	abaca, agave, almond, anise, areca, bambara, bean, brazil, broad bean, buckwheat, canary seed, cashew, castor, cereal, chestnut, chickpea, chicory, cinnamon, clove, cocoa, coconut, coffee, cowpea, fiber, flax, fonio, ginger, gums, hazelnut, hemp, hempseed, hop, jute, jute-like fiber, kapok fiber, kapok seed, karite, kola nut, lentil, linseed, lupin, mate, melon seed, mixed grain, mustard, nutmeg, nut, oats, oilseed, olive, pea, pepper, peppermint, pigeon pea, pimento, pistachio, poppy, pulse, pyrethrum, quinoa, ramie, root, rubber, safflower, sesame, sisal, spice, sugar, taro, tea, tobacco, triticale, tung, vanilla, vetch, walnut, yam, yautia

Figure S1 shows the global distribution of ammonia demand for synthetic nitrogen fertilizers derived for the nitrogen demand in agriculture (**Methods – Section 4.1**). The majority of global demand for ammonia is in China (24%), India (17%) and the United States (9%).

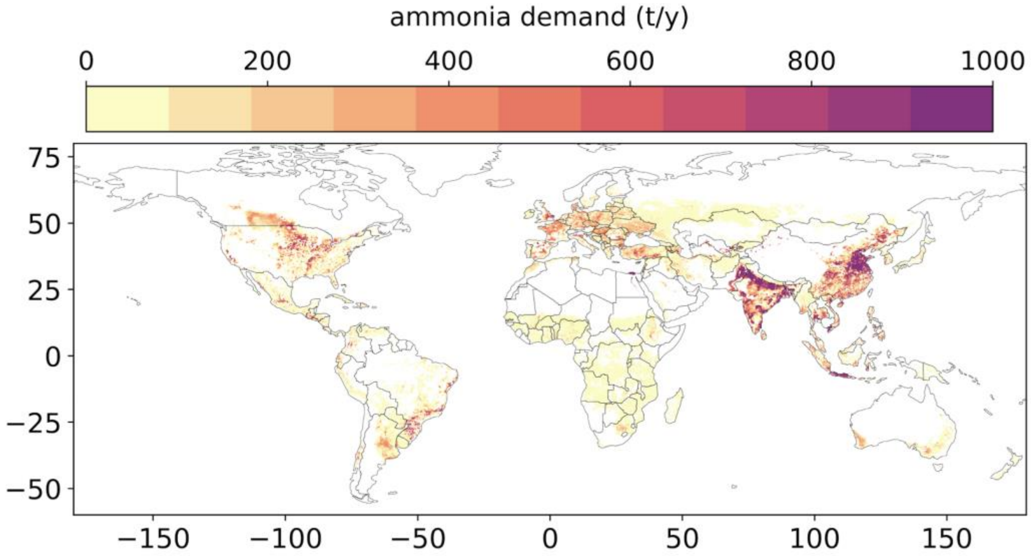

Figure S1. Ammonia demand in 2020. Spatially-explicit distribution of global demand of ammonia for fertilizers production equals 120 Mt/y in 2020, based on the dataset considered. This is the most up-to-date geospatial dataset of current ammonia demand. Maps are created with the Matplotlib and Geopandas packages for Python[28,29].

S.2 Spatially-explicit cost of ammonia

The approach for the calculation of the cost of ammonia (EUR/t_{NH3}) is derived from (**Hockman et al, 2020**)[3], and adapted to data with geospatial resolution, where the electricity price is computed as levelized cost of electricity. We also updated and extended the analysis of the parameters involved in the calculation. We selected this approach with the goal of keeping the calculation with geospatial resolution (the levelized cost of electricity) separated from the scalar parameters assumed for the technology for ammonia production, in the case of electricity supply from the grid. Additionally, due to the presence of assumptions related to the low maturity or low deployment of the technologies considered, this approach allows a straightforward derivation of different cost of ammonia production by assuming different parameters than the values proposed in this study. All the components in the system considered are visualized in the system configurations in **Figure S2**.

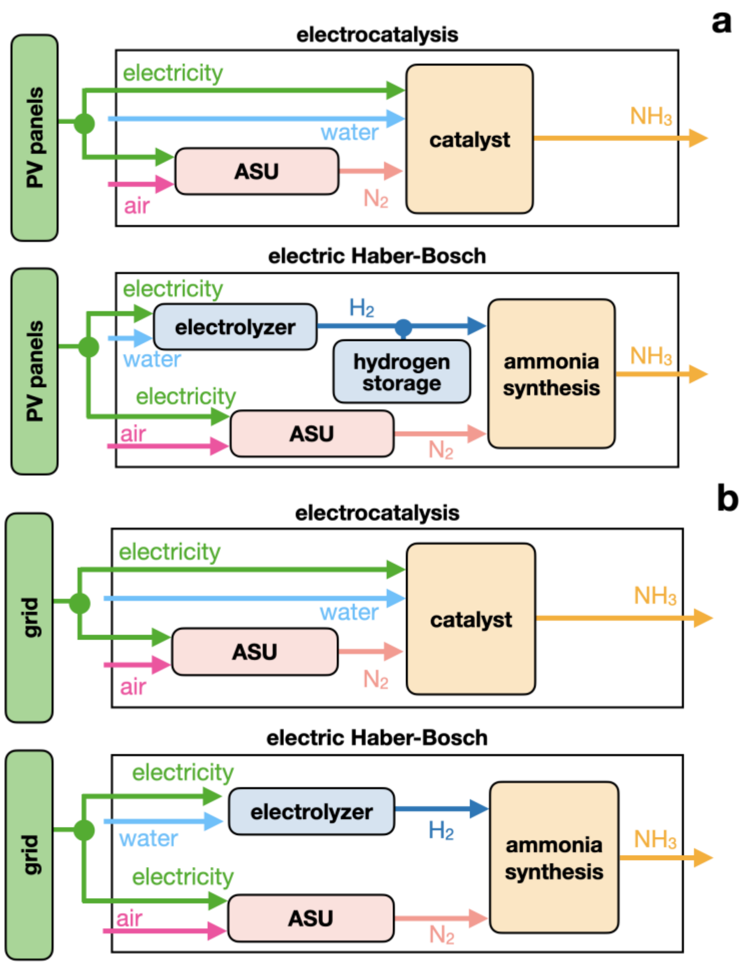

Figure S2. Distributed ammonia conversion systems. This study compares two technologies for ammonia production from electric Haber-Bosch, and electrocatalysis in two configurations: **a** as agrivoltaic systems fed with electricity from solar panels, and **b** as systems fed with electricity from the grid. The agrivoltaic system requires the additional storage of hydrogen for continuous operation of the ammonia synthesis loop, while the operation of this system depends on the capacity factor of the solar panel. Grid-connected systems can run continuously at high-capacity factor and don't need additional storage components.

Levelized cost of electricity

We compute the cost of energy feeding the technologies for ammonia production based on the levelized cost of electricity, c^e_i (EUR/kWh), according to (**Branker et al.**)[4], in every cell i:

$$c^e_i = (C + \sum_{t=1}^{T} O/(1+r)^t)/(\sum_{t=1}^{T}(S_i/F_i\,(1-d)^t/(1+r)^t)) \qquad \forall\ i \in G \qquad (1)$$

with:

- C (EUR/kW) capital expenditure for solar panels,
- O (EUR/kW/y) operation and maintenance fixed costs,
- r (-) discount rate,
- d (-) degradation rate,
- T (y) lifetime of solar panels,
- S_i (kWh/y) solar energy production according to Equation 2,
- F_i (kW) capacity installed according to Equation 3.

The yearly average solar power production, S, is a technology-, time-, space- and weather-dependent parameter. We quantify this parameter with a bottom-up approach, based on a yearly-average geographical discretization at 0.75° × 0.75° grid resolution (or about 80 km × 80 km at the Equator), resampled to 0.083° × 0.083° grid resolution (or about 10 km × 10 km at the Equator). The energy production from solar photovoltaics for all cells, G, is computed as:

$$S_i^{solar} = \eta^{solar}\, I_i\, A_i\, \gamma \qquad \forall\ i \in G \qquad (2)$$

with:

- S_i^{solar} (TWh/y) yearly energy production from solar panels in grid cell i,
- A_i (km^2) cropland soil area in cell i,
- γ (-) ratio of panel area to soil covered area[5],
- η^{solar} (-) conversion efficiency of solar panels[5],
- I_i ($TWh/km^2/y$) yearly average global horizontal irradiation in cell i from (**ESMAP, 2019**)[6].

The installed capacity, F_i (kW), is computed as:

$$F_i = S_i / f_i / 8760 \qquad \forall\ i \in G \qquad (3)$$

with:

- f_i (-) capacity factor in cell i from (**ESMAP, 2019**)[6].

Details of the coefficients involved in the calculation of the levelized cost of electricity are summarized in **Table S2**.

Table S2. Coefficients for calculation of levelized cost of electricity.

symbol	description	values	reference
C (EUR/kW)	capital expenditure for distributed commercial solar panels including cost of balance of plant	2020: 1674.3 (1172.0, 2176.6)<br>2030: 866.4 (606.5, 1126.3)<br>2050: 665.0 (465.5, 864.5)	central value (NREL, 2021)[18]; relative variation compared to central value (IEA, 2021)[19]
O (EUR/kW/y)	fixed operation and maintenance expenses for commercial photovoltaic panels	2020: 18.1 (17.1, 19.0)<br>2030: 11.4 (10.5, 12.4)<br>2050: 9.5 (8.6, 10.5)	(NREL, 2021)[18]
r (-)	nominal discount rate	0.089 (0.05, 0.1)	(NREL, 2021)[18]
I (MWh/y)	yearly-average spatially-explicit solar potential	values change by location	(ESMAP, 2019)[6]
T (y)	solar panels lifetime	2020: 30<br>2030: 35<br>2050: 35	(Terlouw et al., 2022)[20]
f (-)	spatially-explicit capacity factor	values change by location	(ESMAP, 2019)[6]
d (-)	degradation rate	0.005 (0.0036, 0.0064)	(Dupont et al., 2020)[21]
η^{solar} (-)	PV panels conversion efficiency from solar radiation to electricity	0.14	(Amaducci et al., 2018)[5]
A (km^2)	sum of harvested area for 16 major crops	values change by location	(Adalibieke et al., 2023)[1]
γ (-)	ratio of total panel surface to soil surface	0.36	(Amaducci et al., 2018)[5]

Electrocatalysis

The chemical reaction of electrocatalysis is:

$$3\ H_2O + N_2 \rightarrow 2\ NH_3 + 1.5\ O_2 \qquad (4)$$

We derive cost of ammonia production by considering the variable and fixed costs of the systems summarized in **Figure S2** of **Supplementary Information - Section S.2 – Spatially Explicit Cost of Ammonia**. Two configurations are considered for the electricity supply:

a) independent system with electricity fed from agrivoltaic solar panel,
b) grid-connected system.

In case of electricity from agrivoltaic solar panels, the electrolyzer in the electric Haber-Bosch process, and the electrocatalyst, operate at a capacity factor which is the same as the agrivoltaic panel. Since the ammonia synthesis loop requires continuous operation, a storage capacity for hydrogen is assumed between the electrolyzer and ammonia synthesis loop. Differently, we assumed that a continuous operation is possible in case of connection to the grid. In our analysis we considered a system designed for the single target of producing ammonia as fertilizer. However, more complex configurations can involve the use of by-product hydrogen from electrocatalysis (due to a Faradaic efficiency in **Equation S7** lower than 100%), as a fuel for agricultural vehicles, for reconversion into electricity with a fuel-cell, or for heat production.

The total cost of ammonia production is computed as the sum of the variable, v, and fixed costs, w. In case of electricity production from the grid, the fixed costs are independent from the spatial resolution of the analysis, since a constant capacity factor is assumed:

$$c^a_i = v_i + w \qquad (5)$$

with:

- c^a_i (EUR/t_{NH3}): total cost of ammonia production in cell i;
- v_i (EUR/t_{NH3}): variable cost due to the production of ammonia in cell i;
- w (EUR/t_{NH3}): fixed cost of the system.

The variable cost in cell i, v_i, is computed from the enthalpy of reaction in the electrocatalyst, the conversion efficiency, and electricity consumption of the air separation unit. The variable cost is specific to cell i, due to the dependence from the levelized cost of electricity (**Equation S1**):

$$v_i = (e/\eta + e_{ASU})\ c^e_i \qquad (6)$$

where:

- c^e_i (EUR/MWh): levelized cost of electricity from PV in cell i;
- e (MWh/t_{NH3}): enthalpy of reaction of nitrogen electroreduction to ammonia;
- e_{ASU} (MWh/t_{NH3}): energy consumption for operation of air separation unit;
- η (-): conversion efficiency of electrocatalysis.

While for the case of electrolysis, industrial data on the average efficiency of existing technologies are available, electrocatalysis is a technology at low TRL, for which data are only available at laboratory scale.

Accordingly, we derive the efficiency of the conversion from the Faradaic efficiency and the practical potential, from measured data (2020), and the expected performance (2030, 2050):

$$\eta = \eta_f \cdot \eta_v \qquad (7)$$

$$\eta_v = U^{th}/(U^{th} + U^{op}) \qquad (8)$$

where:

- η_f (-): Faradaic efficiency;
- η_v (-): voltage efficiency;
- U^{th} (V): theoretical potential;
- U^{op} (V): overpotential.

The fixed cost is derived from the two components of the system, electrocatalyst and air separation unit (ASU), in addition to the cost of maintenance of the plant:

$$w = c_{catalyst} + c_{oaM} + c_{ASU} \qquad (9)$$

where:

- c_{oaM} (EUR/t_{NH3}): fixed for operation and maintenance;
- c_{ASU} (EUR/t_{NH3}): capex of air separation unit;
- $c_{catalyst}$ (EUR/t_{NH3}): capex of catalyst.

The fixed cost of the electrocatalyst is derived from the cost of electrolyzers, assumed as the closest technology for technological development. The capacity factor assumed in the case of connection to the grid, f, is assumed as constant:

$$c_{catalyst,i} = \tilde{c}_{electrolyzer} /(T \cdot f \cdot 8760 \cdot \eta /(1000 \cdot LHV_{NH3})) \qquad (10)$$

with:

- $\tilde{c}_{electrolyzer}$ (EUR/kW_{el}): capex of reference electrolyzer;
- LHV_{NH3} (MWh/kg): hydrogen lower heating values;
- f (-): capacity factor in case of electricity from the grid;
- T (y): lifetime of electrocatalyst.

In case of electricity production from the agrivoltaic panels, the fixed cost of ammonia production in cell i, c^a_i, is dependent from the spatial resolution of the analysis, due to the assumption of cell specific values:

$$c^a_i = v_i + w_i \qquad (11)$$

In this case, the fixed cost in cell i reads:

$$w_i = c_{catalyst,i} + c_{oaM} + c_{ASU} \qquad (12)$$

The dependence from cell i relates to the consideration of the local capacity factor f_i:

$$c_{catalyst,i} = \tilde{c}_{electrolyzer} /(T \cdot f_i \cdot 8760 \cdot \eta /(1000 \cdot LHV_{NH3})) \quad (13)$$

with:

- f_i (-): capacity factor in case of electricity from agrivoltaics solar panels in cell i.

Details of the coefficients involved in the calculation of the cost of ammonia from electrocatalysis are summarized in **Table S3**.

We assumed a Faradaic efficiency (η_f in Equation 7) varying between 50% (2020), to 60% (2030) with an overpotential of 0.7 V, obtained from data presented in the Supplementary Information of reference literature[7] and referring to d-metal and perovskite as catalytic materials. These assumptions correspond to an energy efficiency of 31% and 38% in 2020 and 2030, respectively. Instead, data for 2050 refers to the target set by DOE[8] of 90% Faradaic efficiency with 0.7 V overpotential, corresponding to 56% energy efficiency. The reported current densities for this range of energy efficiencies[7] in 2020 and 2030 is around 1 mA/cm^2, while for 2050 the US Department of Energy requires current densities greater than 500 mA/cm^2. Concerning the lifetime of the system, due to the low TRL of the electrocatalyst, we took the same assumption for electrocatalyst as for the electrolyzer in the electric Haber-Bosch.

Table S3. Coefficients for calculation of ammonia production cost from electrocatalysis.

symbol	description	values	Reference
e (MWh/t_{NH3})	enthalpy of reaction of nitrogen electroreduction	5.675	(D'Angelo et al., 2023)[22]
e_{ASU} (MWh/t_{NH3})	energy consumption for operation of the air separation unit	0.299	(D'Angelo et al., 2023)[22]
U^{th} (V)	theoretical cell voltage	1.17	(D'Angelo et al., 2023)[22]
U^{op} (V)	cell overpotential	0.3 - electrode<br>0.1 - ohmic drop	(D'Angelo et al., 2023)[22]
η_f (-)	Faradaic efficiency	2020: 0.5 (0.4, 0.6)<br>2030: 0.6 (0.5, 0.7)<br>2050: 0.9 (0.85, 0.95)	2020, 2030<br>(Martín et al., 2019)[7]<br>2050<br>(DOE, 2016)[8]
c_{oaM} (-)	fixed operation and maintenance cost	2% of $c_{catalyst}$	(Terlouw et al., 2022)[14]
c_{ASU} (-)	capex of air separation unit (ASU)	7% of $c_{catalyst}$	(Hochman et al., 2020)[3]
$\tilde{c}_{electrolyzer}$ (EUR/kW_{el})	capex of PEM electrolyzer	2020: 1150 (935, 1266)<br>2030: 717 (500, 935)<br>2050: 397 (149, 510)	(Everall et al, 2021)[23]
$m_{reaction}$ (t_{NH3}/t_{H2})	stoichiometric ratio of hydrogen conversion into ammonia	34/6 = 5.67	(Hochman et al., 2020)[3]
LHV_{NH3} (MWh/kg)	ammonia lower heating value	5.17	(Smith et al., 2020)[24]
T (y)	system lifetime	30	(Nayak-Luke et al., 2020)[9]
f (-)	capacity factor electrolyzer in case of electricity from the grid	90%	This study

Electric Haber-Bosch

The production of ammonia in the electric Haber-Bosch technology involves two reactions, in the electrolysis and in the Haber-Bosch ammonia synthesis loop:

$$H_2O \rightarrow H_2 + 0.5\ O_2 \qquad \text{(water electrolysis)} \qquad (14)$$

$$3\ H_2 + N_2 \rightarrow 2\ NH_3 \qquad \text{(ammonia synthesis)} \qquad (15)$$

The total cost of ammonia, in the case with electricity supply from the grid is computed as:

$$c^a_i = v_i + w \qquad (16)$$

with:

- c^a_i (EUR/t_{NH3}): total cost of ammonia production in cell i;
- v_i (EUR/t_{NH3}): variable cost due to the production of ammonia in cell i;
- w (EUR/t_{NH3}): fixed cost independent from the spatial resolution.

The variable cost, v, is computed from the enthalpy of reaction in the electrocatalyst, the conversion efficiency, and electricity consumption of the air separation unit. The variable cost is specific to cell i, due to the dependence from the levelized cost of electricity (**Equation S1**):

$$v_i = c^e_i \cdot (e_{eletrolyzer} / m_{reaction} / \eta_{electrolyzer} + e_{NH3\ synthesis} + e_{ASU}) \qquad (17)$$

with:

- c^e_i (EUR/MWh): levelized cost from PV in cell i;
- $e_{eletrolyzer}$ (MWh/t_{H2}): lower heating value of hydrogen;
- $\eta_{electrolyzer}$ (-): electrolyzer efficiency with respect to the lower heating value of hydrogen;
- $e_{NH3\ synthesis}$ (MWh/t_{NH3}): energy consumption for ammonia synthesis from hydrogen feedstock;
- e_{ASU} (MWh/t_{NH3}): energy consumption for operation of the air separation unit.

The fixed cost is derived from electrolyzer, the Haber-Bosch synthesis loop, the air separation unit (ASU), in addition to the cost of maintenance of the plant:

$$w = c_{electrolyzer} + c_{NH3\ synthesis} + c_{oaM} + c_{ASU} + s \qquad (18)$$

$$c_{electrolyzer} = \tilde{c}_{electrolyzer} / (T \cdot f \cdot 8760 \cdot \eta_{electrolyzer} \cdot m_{reaction} / (1000 \cdot LHV_{H2})) \qquad (19)$$

with:

- $c_{NH3\ synthesis}$ (EUR/t_{NH3}): fixed cost of ammonia synthesis;
- c_{oaM} (EUR/t_{NH3}): fixed for operation and maintenance;
- c_{ASU} (EUR/t_{NH3}): capex of air separation unit;
- s (EUR/t_{NH3}): cost of storage;
- $\tilde{c}_{electrolyzer}$ (EUR/kW_{el}): capex of electrolyzer;
- $m_{reaction}$ (-): stoichiometric ratio of hydrogen conversion into ammonia;

- $\eta_{electrolyzer}$ (-): conversion efficiency electrolyzer;
- LHV_{H2} (MWh/kg): hydrogen lower heating values;
- T (y): lifetime of system.

In case of electricity production from the agrivoltaic panels, the fixed cost is dependent from the spatial resolution of the analysis, due to the assumption of cell specific values:

$$c^a_i = v_i + w_i \qquad (20)$$

In this case, the fixed cost reads:

$$w_i = c_{electrolyzer,i} + c_{NH3\ synthesis} + c_{oaM} + c_{ASU} + s \qquad (21)$$

$$c_{electrolyzer,i} = \tilde{c}_{electrolyzer}/(T \cdot f_i \cdot 8760 \cdot \eta_{electrolyzer} \cdot m_{reaction} /(1000 \cdot LHV_{H2})) \qquad (22)$$

with:

- f_i (-): capacity factor in case of electricity from agrivoltaics solar panels in cell i.

Details of the coefficients involved in the calculation of the cost of ammonia from electric Haber-Bosch are summarized in **Table S4**.

We considered a lifetime of 30 years for the electrolyzer, in agreement with literature[10], and coherently with a design lifetime for PEM electrolyzers of 60000 operating hours[10,11], with a capacity factor equal to the global average of 20% from the agrivoltaic system considered in study. We assumed a full replacement of the system at the end of the lifetime, i.e., including the full stack replacement.

Table S4. Coefficients for calculation of ammonia production cost from electric Haber-Bosch.

symbol	description	values	reference
$e_{eletrolyzer}$ (MWh/t_{H2})	lower heating value of hydrogen	33.34	(D'Angelo et al., 2023)[22]
e_{ASU} (MWh/t_{NH3})	energy consumption for operation of the air separation unit	0.299	(D'Angelo et al., 2023)[22]
$e_{NH3\ synthesis}$ (MWh/t_{NH3})	energy consumption for ammonia synthesis from hydrogen feedstock	2020: 5.33<br>2030: 3.44<br>2050: 1.54	(Smith et al., 2020)[17]
$\eta_{electrolyzer}$ (-)	PEM electrolyzer conversion efficiency with respect to the lower heating value of hydrogen	2020: 0.62 (0.54, 0.67)<br>2030: 0.66 (0.53, 0.72)<br>2050: 0.71 (0.7, 0.72)	(Everall et al, 2021)[23]
$\tilde{c}_{electrolyzer}$ (EUR/kW_{el})	capex of PEM electrolyzer	2020: 1150 (935, 1266)<br>2030: 717 (500, 935)<br>2050: 397 (149, 510)	(Everall et al, 2021)[23]
$c_{NH3\ synthesis}$ (EUR/t_{NH3})	ammonia synthesis capex (derivation from production of 100 t/d and conversion USD to EUR based on 2021 average)	32.2	(Wang et al, 2021)[25]<br>(ECB, 2021)[26]
c_{oaM} (-)	fixed operation and maintenance cost	2% of ($c_{electrolyzer} + c_{NH3\ synthesis}$)	(Terlouw et al., 2022)[20]
c_{ASU} (-)	capex of air separation unit (ASU)	39% of ($c_{NH3\ synthesis}$)	(Ikäheimo, 2018)[27]
s (-)	capex hydrogen storage technology	31% (19%, 43%) of ($c_{electrolyzer} + c_{NH3\ synthesis} + c_{oaM}$)	(Nayak-Luke et al., 2020)[9]
T (-)	system lifetime	30	(Nayak-Luke et al., 2020)[9]
f (-)	capacity factor electrolyzer in case of electricity from the grid	90%	This study
$m_{reaction}$ (t_{NH3}/t_{H2})	stoichiometric ratio of hydrogen conversion into ammonia	34/6 = 5.67	(Hochman et al., 2020)[3]

Spatially-explicit cost of ammonia

Figure S3 shows the global distribution of the spatially-explicit cost of ammonia production computed for electric Haber-Bosch and electrocatalysis under different cost assumptions in 2020, 2030 and 2050, representative of the technological development. The cost of ammonia production in **Figure S3 a-c** and **g-i** is representative of a system connected to the grid, **Figure S3 d-f** and **j-l** of a system connected to the grid. This second case does not require any hydrogen storage to guarantee the continuous operation of the ammonia synthesis loop in the electric Haber-Bosch technology. Additionally, the operation of the two technologies is assumed to be continuous and is not affected by the capacity factor of the solar panel. Since no cropland is present above 60° N latitude, fertilizers demand is equal to zero in those high latitude regions, which are excluded from our analysis.

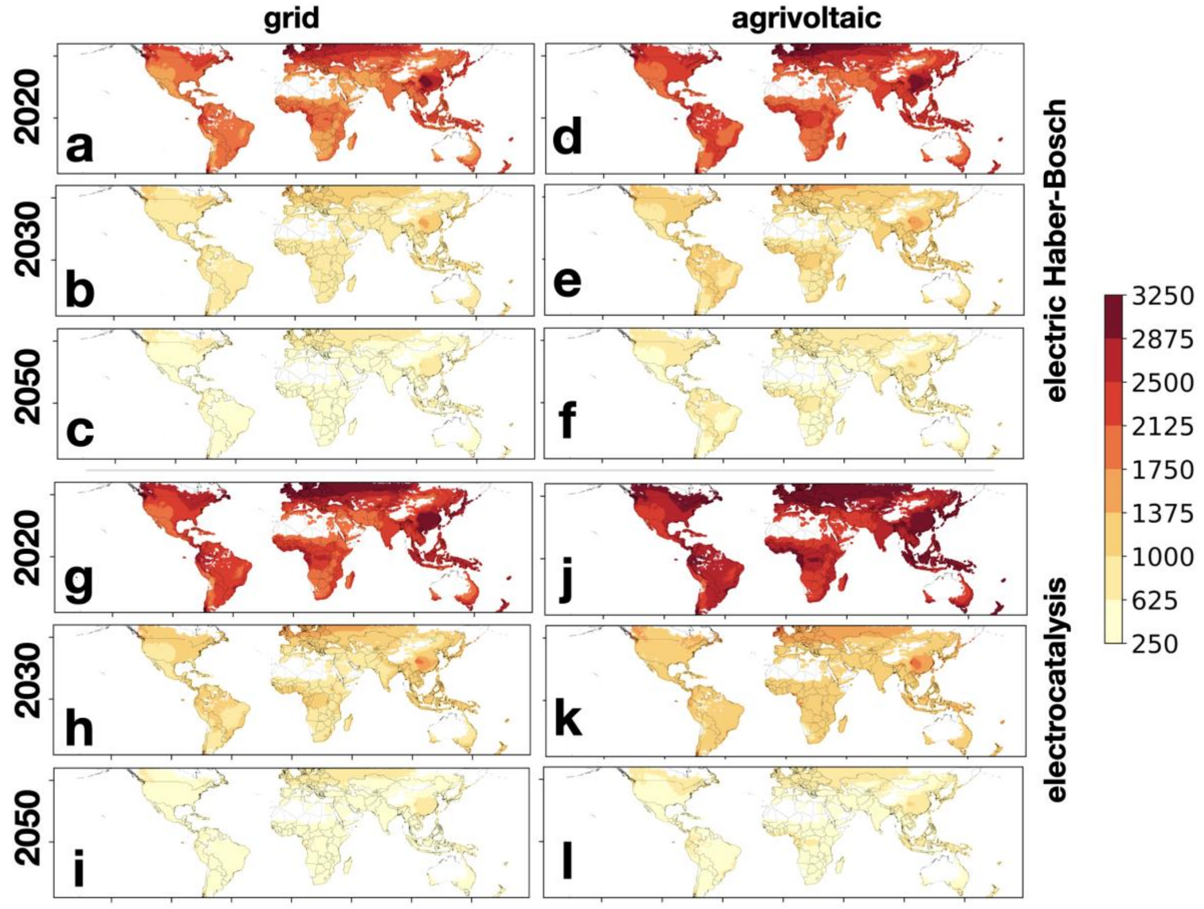

Figure S3. Spatially-explicit cost of ammonia production. Cost of ammonia production from **a-f** electric Haber-Bosch, and **g-l** electrocatalysis. Two systems are considered: **a-c** and **g-i** from connection to the grid, and **d-f** and **j-l** fed with intermittent power production from agrivoltaic solar panels.

Cost breakdown

The cost breakdown analysis described in this section is shown in **Figure S4** in the two configurations considered for electric Haber-Bosch (**Figure S4 a, c**) and electrocatalysis (**Figure S4 b, d**) for cost assumptions in 2020, 2030, and 2050. The grid configuration corresponds to the case of electricity supply from the electrical grid, while the agrivoltaic configuration corresponds to electricity supply from solar panels. Accordingly, the cost of the agrivoltaic configuration reflects the lower capacity factor of solar panels, and the need of hydrogen storage to guarantee the operation of the ammonia synthesis loop in the electric Haber-Bosch. The impact of the capacity factor is reflected in the fixed cost of the electrolyzer (panel of fixed costs in **Figure S4 c** compared to **Figure S4 a**) and the electrocatalyst (panel of fixed costs in **Figure S4 d** compared to **Figure S4 b**), leading to a fivefold increase in fixed costs. Hydrogen storage determines an additional 22% increase in the fixed costs. The largest fraction of fixed and variables costs is associated to the capital expenditure of the electrolyzer and the electrocatalyst. For electric Haber-Bosch, a substantial portion of the costs is associated to the synthesis of ammonia from hydrogen, representing 8-20% of the fixed costs, and between 15-35% of the variable costs (**Figure S4 a, c**). While for electrocatalysis, electricity demand represents approximately 98% of the total variable costs (**Figure S4 b, d**). For the two technologies considered, variable costs represent the largest fraction of the total costs. The fraction of variables costs is between 81- 86% for electrocatalysis, in case of electricity from agrivoltaic system, 96 - 97% in case of electricity from the grid. For electric Haber-Bosch, the fraction of variables costs is 77 - 83%, in case of electricity from agrivoltaic system, 90 - 95% in case of electricity from the grid.

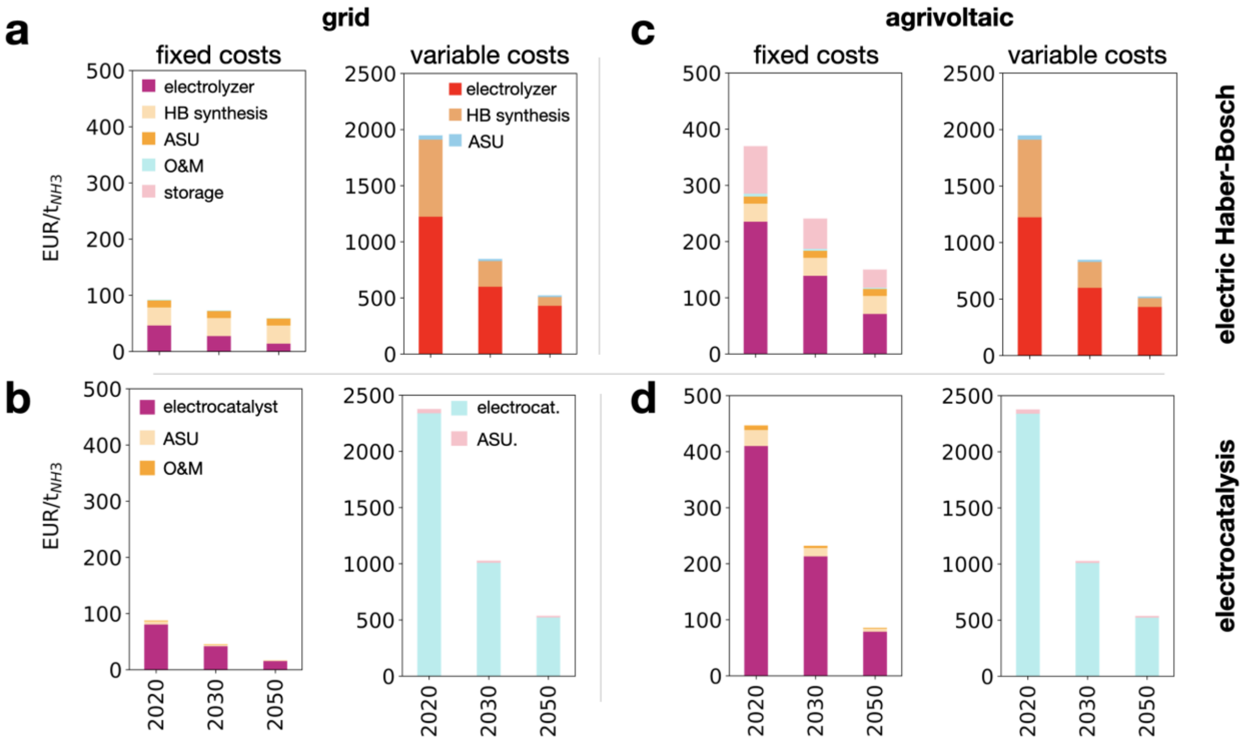

Figure S4. Breakdown of the cost of ammonia production. The figure reports the average values of the spatially-explicit ammonia production costs with spatial resolution shown in **Figure S3**. Panels **a** and **b** refer to ammonia production from electric Haber-Bosch and electrocatalysis, respectively, with electricity fed from the grid in continuous operation. Panels **c** and **d** refer to ammonia production from electric

Haber-Bosch and electrocatalysis, respectively, with electricity fed from an intermittent agrivoltaic-based system.

S.3 Ammonia market price

To assess the cost-competitiveness of the technologies considered in our analysis, we compare the technology-specific cost of ammonia with the variation of the historical ammonia market price from the World Bank commodity price database[12]. As the most common form of ammonia-based fertilizer, we consider urea as product representative of the fertilizer price. Data from the World Bank commodity price database is elaborated from the granular urea free on board (f.o.b.) Black Sea market price. Price quotes are representative of the current market conditions as perceived by selected buyers and sellers involved in large transactions, and do not necessarily reflect actual transactions[12,13].

Specifically, we consider the yearly-average real urea market price (EUR/t_{urea}) between 2008 – 2022 converted into ammonia market price (EUR/$t_{ammonia}$) based on the mass balance of urea production (0.57 $t_{ammonia}$/t_{urea}):

$$2\ NH_3\ (17\ g/mol) + CO_2\ (44\ g/mol) \rightarrow CH_4N_2O\ (60\ g/mol) + H_2O\ (18\ g/mol)$$

Results are presented in EUR, with conversion from USD based on on historical exchange reference rates[14]. From the historical distribution, we derive the price statistics used in **Figure 1** of the main text, here summarized in **Figure S5** and **Table S5**.

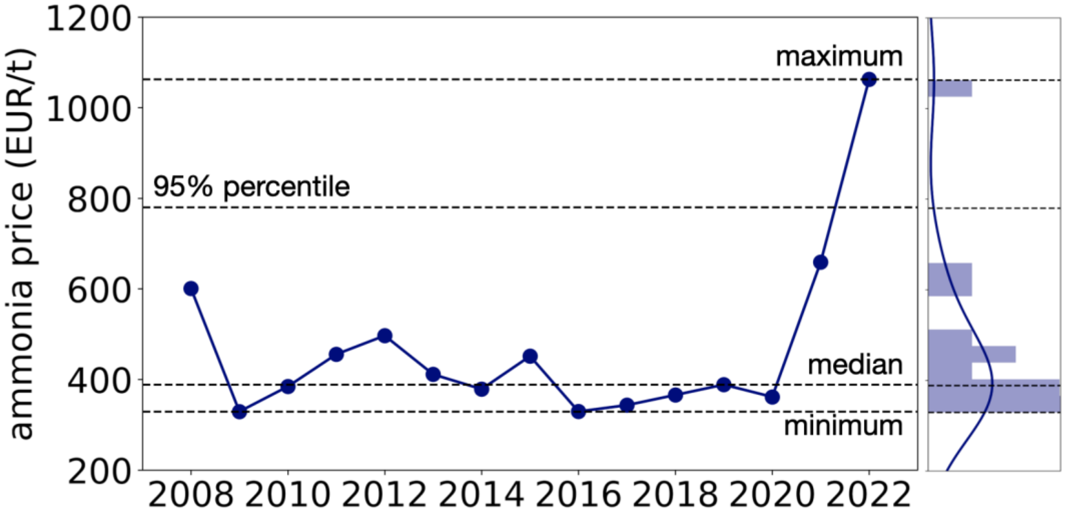

Figure S5. Historical ammonia market price over the last 15 years between 2008 and 2022 (EUR/$t_{ammonia}$). Data elaborated from the urea real market price of the World Bank commodity price database[12].

Table S5. Statistics from ammonia price distribution (EUR/t). The first row reports the statistics of the ammonia spot market price over the last 15 years. The second row reports the sum of the ammonia spot market price, assumed as production cost in the study, with the extreme scenario of additional fertilizer transport cost doubling the fertilizer price from the production stage.

	**minimum**	**median**	**95% percentile**	**maximum**
**production (spot market)**	329	389	780	1063
**production + transport (upper bound cost of transport)**	658	777	1560	2125

S.4 Sensitivity analysis

We carry out a sensitivity analysis to quantify the impact of uncertainty on some of the main parameters involved in the calculation of the cost of ammonia (**Methods – Section 4.3**) based on the total cost of production and the Sobol indices. The sources of uncertainty are both the global geographical coverage of the analysis and the temporal coverage of the scenarios considered (from 2020 to 2050). While in this study we used a simplified model of the capital and operating costs, local conditions and uncertainties (like variable energy supply, temperature management) should be taken into consideration for more accurate analyses of decentralized production[15,16]. **Table S2**, **S3** and **S4** reports the reference values and the range of variation of the parameters involved in the calculation. **Figure S6 (a,b)** indicate that, both in case of ammonia production from electric Haber-Bosch and electrocatalysis, the main parameters affecting the median of the global distribution of ammonia is the local cost of electricity, dependent on the capital expenditures and the discount rate of solar photovoltaics. For electric Haber-Bosch, the electrolyzer efficiency is one of the parameters affecting the most the cost of ammonia production. For electrocatalysis, the median of the cost of ammonia is mainly affected by the faradaic efficiency (**Figure S6 (b)**) whose range of variation is large in 2020 and 2030, due to the low maturity of the technology (TRL 1-3)[17].

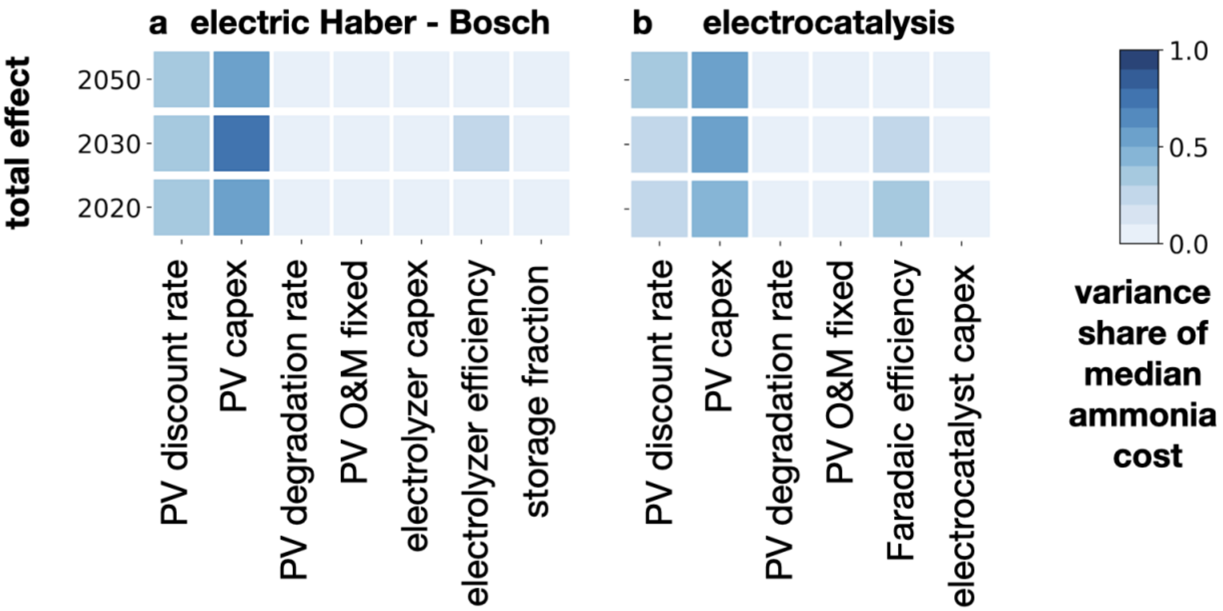

Figure S6. Impact of uncertainty on median of the global distribution of the cost of ammonia. The main parameters affecting the median of the cost of ammonia are the capital expenditures of photovoltaic panels and the discount rate over the lifetime of the panels which determine the local levelized cost of electricity. Additionally, the median of the cost of ammonia in case of electrocatalysis is mainly affected by the faradaic efficiency of the technology (η_f in **Equation S7** in **Suppl. Inf. – Section S.1**).

SUPPLEMENTARY REFERENCES

1. Adalibieke, W., Cui, X., Cai, H., You, L. and Zhou, F. Global crop-specific nitrogen fertilization dataset in 1961–2020. *Scientific Data* **10(1)**, 617 (2023).
2. FAO. FAOSTAT Fertilizers by Nutrient. http://www.fao.org/faostat/en/#data/RFN (2022).
3. Hochman, G., Goldman, A.S., Felder, F.A., Mayer, J.M., Miller, A.J., Holland, P.L., Goldman, L.A., Manocha, P., Song, Z. & Aleti, S., 2020. Potential economic feasibility of direct electrochemical nitrogen reduction as a route to ammonia. *ACS sustainable chemistry & engineering* **8(24)**, 8938-8948.
4. Branker, K., Pathak, M.J.M. & Pearce, J.M. A review of solar photovoltaic levelized cost of electricity. *Renewable and sustainable energy reviews* **15(9)**, 4470-4482 (2011).
5. Amaducci, S., Yin, X. & Colauzzi, M. Agrivoltaic systems to optimise land use for electric energy production. *Applied energy* **220**, 545-561 (2018).
6. ESMAP. Global Solar Atlas 2.0. Technical Report, World Bank. https://globalsolaratlas.info/map (2019).
7. Martín, A.J., Shinagawa, T. & Pérez-Ramírez, J. Electrocatalytic reduction of nitrogen: from Haber-Bosch to ammonia artificial leaf. *Chem* **5(2)**, 263-283 (2019).
8. DOE. Advanced Research Projects Agency-Energy-US Department of Energy, Renewable Energy to Fuels Through Utilization of Energy Dense Liquids (REFUEL) Program Overview. https://arpa-e.energy.gov/sites/default/files/documents/files/REFUEL_ProgramOverview.pdf (2016).
9. Nayak-Luke, R.M. & Bañares-Alcántara, R. Techno-economic viability of islanded green ammonia as a carbon-free energy vector and as a substitute for conventional production. *Energy & Environmental Science* **13(9)**, 2957-2966 (2020).
10. Schmidt, O., Gambhir, A., Staffell, I., Hawkes, A., Nelson, J. & Few, S. Future cost and performance of water electrolysis: An expert elicitation study. *International journal of hydrogen energy* **42(52)**, 30470-30492 (2017).
11. Buttler, A. and Spliethoff, H. Current status of water electrolysis for energy storage, grid balancing and sector coupling via power-to-gas and power-to-liquids: A review. *Renewable and Sustainable Energy Reviews* **82**, 2440-2454 (2018).
12. Ha, Jongrim, M. Ayhan Kose, & Franziska Ohnsorge. One-Stop Source: A Global Database of Inflation.Policy. World Bank, Research Working Paper 9737 (2021).
13. Green Markets - A Bloomberg Company. Fertilizer Pricing Data Feeds. https://fertilizerpricing.com/data-services/custom-data-feeds/
14. Eurostat, Euro foreign exchange reference rate, https://www.ecb.europa.eu/stats/policy_and_exchange_rates/euro_reference_exchange_rates/html/eurofxref-graph-usd.en.html
15. Verleysen, K., Coppitters, D., Parente, A., De Paepe, W. & Contino, F. How can power-to-ammonia be robust? Optimization of an ammonia synthesis plant powered by a wind turbine considering operational uncertainties. *Fuel* **266(117049)**, (2020).
16. Verleysen, K., Parente, A. & Contino, F. How sensitive is a dynamic ammonia synthesis process? global sensitivity analysis of a dynamic Haber-Bosch process (for flexible seasonal energy storage). *Energy* **232(121016)**, (2021).
17. Smith, C., Hill, A.K. & Torrente-Murciano, L. Current and future role of Haber–Bosch ammonia in a carbon-free energy landscape. *Energy & Environmental Science* **13(2)**, 331-344 (2020).
18. National Renewable Energy Laboratory. Annual Technology Baseline. National Renewable Energy Laboratory https://atb.nrel.gov/ (2021).
19. International Energy Agency, Net Zero by 2050. https://www.iea.org/reports/net-zero-by-2050 (2021).
20. Terlouw, T., Bauer, C., McKenna, R. & Mazzotti, M. Large-scale hydrogen production via water electrolysis: a techno-economic and environmental assessment. *Energy & Environmental Science* **15(9)**, 3583-3602 (2022).
21. Dupont, E., Koppelaar, R. & Jeanmart, H. Global available solar energy under physical and energy return on investment constraints. *Applied Energy* **257**, 113968 (2020).

22. D'Angelo, S.C., Martín, A.J., Guillén-Gosálbez, G. & Pérez-Ramírez, J., 2023. The Environmental Feasibility of Decentralised Solar Ammonia. *Chimia* **77(3)**, 150-153.
23. Everall, J., & Ueckerdt, F. Electrolyser CAPEX and efficiency data for: Potential and risks of hydrogen-based e-fuels in climate change mitigation. https://doi.org/10.5281/zenodo.4619892 (2021).
24. Smith, C., Hill, A.K. & Torrente-Murciano, L. Current and future role of Haber–Bosch ammonia in a carbon-free energy landscape. *Energy & Environmental Science* **13(2)**, 331-344 (2020).
25. Wang, M., Khan, M.A., Mohsin, I., Wicks, J., Ip, A.H., Sumon, K.Z., Dinh, C.T., Sargent, E.H., Gates, I.D. & Kibria, M.G., 2021. Can sustainable ammonia synthesis pathways compete with fossil-fuel based Haber–Bosch processes? *Energy & Environmental Science* **14(5)**, 2535-2548.
26. ECB. https://www.ecb.europa.eu/stats/policy_and_exchange_rates/euro_reference_exchange_rates/html/eurofxref-graph-usd.en.html (2021).
27. Ikäheimo, J., Kiviluoma, J., Weiss, R. & Holttinen, H. Power-to-ammonia in future North European 100% renewable power and heat system. *International Journal of Hydrogen Energy* **43(36)**, 17295-17308 (2018).
28. Hunter, J. D. Matplotlib: A 2D Graphics Environment. *Computing in Science & Engineering* **9(3)**, 90-95. https://matplotlib.org/stable/index.html (2007).
29. Jordahl, K. et al. geopandas/geopandas: v0.8.1. Zenodo. http://doi.org/10.5281/zenodo.3946761 (2020).
