# Supplementary material for: Cost-competitive decentralized ammonia fertilizer production can increase food security
Source: Nat Food. 2024 May 16;5(6):469–79. doi: 10.1038/s43016-024-00979-y (PMC11199140; doi:10.1038/s43016-024-00979-y)
Supplement: Supplementary file 2 — Reporting Summary [file 43016_2024_979_MOESM2_ESM.pdf]

Corresponding author(s): Davide Tonelli
Lorenzo Rosa

Last updated by author(s): Apr 6, 2024

Reporting Summary

Nature Portfolio wishes to improve the reproducibility of the work that we publish. This form provides structure for consistency and transparency in reporting. For further information on Nature Portfolio policies, see our Editorial Policies and the Editorial Policy Checklist.

Statistics

For all statistical analyses, confirm that the following items are present in the figure legend, table legend, main text, or Methods section.

n/a	Confirmed	
☒	☐	The exact sample size (*n*) for each experimental group/condition, given as a discrete number and unit of measurement
☒	☐	A statement on whether measurements were taken from distinct samples or whether the same sample was measured repeatedly
☒	☐	The statistical test(s) used AND whether they are one- or two-sided<br>*Only common tests should be described solely by name; describe more complex techniques in the Methods section.*
☒	☐	A description of all covariates tested
☒	☐	A description of any assumptions or corrections, such as tests of normality and adjustment for multiple comparisons
☐	☒	A full description of the statistical parameters including central tendency (e.g. means) or other basic estimates (e.g. regression coefficient) AND variation (e.g. standard deviation) or associated estimates of uncertainty (e.g. confidence intervals)
☒	☐	For null hypothesis testing, the test statistic (e.g. *F*, *t*, *r*) with confidence intervals, effect sizes, degrees of freedom and *P* value noted<br>*Give P values as exact values whenever suitable.*
☒	☐	For Bayesian analysis, information on the choice of priors and Markov chain Monte Carlo settings
☒	☐	For hierarchical and complex designs, identification of the appropriate level for tests and full reporting of outcomes
☒	☐	Estimates of effect sizes (e.g. Cohen's *d*, Pearson's *r*), indicating how they were calculated

Our web collection on statistics for biologists contains articles on many of the points above.

Software and code

Policy information about availability of computer code

Data collection	The raw data used in this study are from open access scientific literature and no software was used for data collection.
Data analysis	Custom made codes were used for data elaboration, analysis and visualization. All the scripts are written in Python 3.9.12. Maps were created with Matplotlib and Geopandas packages. All the codes are stored in the open access Zenodo repository http://doi.org/10.5281/zenodo.8155141

For manuscripts utilizing custom algorithms or software that are central to the research but not yet described in published literature, software must be made available to editors and reviewers. We strongly encourage code deposition in a community repository (e.g. GitHub). See the Nature Portfolio guidelines for submitting code & software for further information.

Data

Policy information about availability of data

All manuscripts must include a data availability statement. This statement should provide the following information, where applicable:

- Accession codes, unique identifiers, or web links for publicly available datasets
- A description of any restrictions on data availability
- For clinical datasets or third party data, please ensure that the statement adheres to our policy

Data are available from the open access repository http://doi.org/10.5281/zenodo.8155141 . The folder "input_data" contains the scripts used to process the raw input data of the study. The subfolder "input_raster" contains the raster files with spatially-explicit resolution at global scale upon which the study is built. The raw

data used in the study come from open-source datasets:
- Historical fertilizer prices: 47. Ha, Jongrim, M. Ayhan Kose, & Franziska Ohnsorge. One-Stop Source: A Global Database of Inflation Policy. World Bank Research Working Paper 9737 (2021).
- Spatially-explicit nitrogen demand in 2020: 42. Adalibieke, W., Cui, X., Cai, H., You, L. & Zhou, F. Global crop-specific nitrogen fertilization dataset in 1961–2020. Scientific Data 10(1), 617 (2023).
- Spatially-explicit solar irradiation: ESMAP. Global Solar Atlas 2.0. Technical Report, World Bank. https://globalsolaratlas.info/map (2019).

Research involving human participants, their data, or biological material

Policy information about studies with human participants or human data. See also policy information about sex, gender (identity/presentation), and sexual orientation and race, ethnicity and racism.

Reporting on sex and gender	No human research participant was involved in this work.
Reporting on race, ethnicity, or other socially relevant groupings	No human research participant was involved in this work.
Population characteristics	No human research participant was involved in this work.
Recruitment	No human research participant was involved in this work.
Ethics oversight	No human research participant was involved in this work.

Note that full information on the approval of the study protocol must also be provided in the manuscript.

Field-specific reporting

Please select the one below that is the best fit for your research. If you are not sure, read the appropriate sections before making your selection.

☐ Life sciences ☐ Behavioural & social sciences ☒ Ecological, evolutionary & environmental sciences

For a reference copy of the document with all sections, see nature.com/documents/nr-reporting-summary-flat.pdf

Ecological, evolutionary & environmental sciences study design

All studies must disclose on these points even when the disclosure is negative.

Study description	The study relies on input data from open source scientific publications and do not involve any laboratory measurements or field work.
Research sample	The spatially-explicit high-resolution research data used for the analysis comes the scientific publication:<br>Adalibieke, W., Cui, X., Cai, H., You, L. and Zhou, F. Global crop-specific nitrogen fertilization dataset in 1961–2020. Scientific Data 10(1), 617 (2023).<br>These data provide deterministic estimations of the nutrients required for crops production under different scenarios.<br><br>Additionally, the calculation of the spatially-explicit high-resolution local cost of ammonia production is derived from cost assumptions based on literature and the solar irradiation from:<br>- ESMAP. Global Solar Atlas 2.0. Technical Report, World Bank. https://globalsolaratlas.info/map (2019).
Sampling strategy	The spatially-explicit data comes from deterministic estimations, based on well defined assumptions.
Data collection	All the data comes from open-source documented literature.
Timing and spatial scale	This point does not apply since the data used comes from numerical calculations.
Data exclusions	No data correction was introduced beyond the calculations stated in the article.
Reproducibility	All the calculations in the article are reproducible based on the codes in the folder open-access.
Randomization	The only allocations of data within this study is the aggregation of data at pixel-level into countries and continents. Data allocation, reproducible with the shared codes, is based on polygons describing the shape of single countries and the pixels contained within the polygons.
Blinding	This point does not apply since the data used comes from numerical calculations.

Did the study involve field work? ☐ Yes ☒ No

Reporting for specific materials, systems and methods

We require information from authors about some types of materials, experimental systems and methods used in many studies. Here, indicate whether each material, system or method listed is relevant to your study. If you are not sure if a list item applies to your research, read the appropriate section before selecting a response.

Materials & experimental systems

n/a	Involved in the study	
☒	☐	Antibodies
☒	☐	Eukaryotic cell lines
☒	☐	Palaeontology and archaeology
☒	☐	Animals and other organisms
☒	☐	Clinical data
☒	☐	Dual use research of concern
☒	☐	Plants

Methods

n/a	Involved in the study	
☒	☐	ChIP-seq
☒	☐	Flow cytometry
☒	☐	MRI-based neuroimaging

Plants

Seed stocks	n/a
Novel plant genotypes	n/a
Authentication	n/a
